# Construction of a guide-RNA for site-directed RNA mutagenesis utilising intracellular A-to-I RNA editing

**DOI:** 10.1038/srep41478

**Published:** 2017-02-02

**Authors:** Masatora Fukuda, Hiromitsu Umeno, Kanako Nose, Azusa Nishitarumizu, Ryoma Noguchi, Hiroyuki Nakagawa

**Affiliations:** 1 Department of Chemistry, Faculty of Science, Fukuoka University, 8-19-1 Nanakuma, Jonan-ku, Fukuoka, Fukuoka 814-0180, Japan; 2 Department of Earth System Science, Faculty of Science, Fukuoka University, 8-19-1 Nanakuma, Jonan-ku, Fukuoka, Fukuoka, 814-0180, Japan

## Abstract

As an alternative to DNA mutagenesis, RNA mutagenesis can potentially become a powerful gene-regulation method for fundamental research and applied life sciences. Adenosine-to-inosine (A-to-I) RNA editing alters genetic information at the transcript level and is an important biological process that is commonly conserved in metazoans. Therefore, a versatile RNA-mutagenesis method can be achieved by utilising the intracellular RNA-editing mechanism. Here, we report novel guide RNAs capable of inducing A-to-I mutations by guiding the editing enzyme, human adenosine deaminase acting on RNA (ADAR). These guide RNAs successfully introduced A-to-I mutations into the target-site, which was determined by the reprogrammable antisense region. In ADAR2-over expressing cells, site-directed RNA editing could also be performed by simply introducing the guide RNA. Our guide RNA framework provides basic insights into establishing a generally applicable RNA-mutagenesis method.

Genetic engineering technologies for regulating intracellular target-gene functions and/or expression have been widely used in basic research and medicinal and therapeutic applications^1^. The purpose of genetic engineering methodologies is frequently to regulate the function of intracellular proteins involved in biological processes of interest. Recently, several genome-editing technologies^1^,^2^,^3^,^4^,^5^ have made it possible to manipulate target-genomic information. In addition to programmable regulation, the most significant feature of genome alteration is that it can provide a permanent change to the targeted cellular gene. While this permanent effect can effectively control target-protein features, such methods pose health risks if an error occurs^6^.

In contrast to DNA, messenger RNA (mRNA) is a transient cellular molecule. mRNAs possess genetic information that determines the functions and expression levels of the encoded proteins. Hence, RNA-targeted genetic manipulation is capable of controlling target-protein functions, similar to genome editing, without the risk of damaging the original genomic information. Small-interfering RNAs (siRNAs)^7^ and microRNAs (miRNA)^8^ have been commonly used to target intracellular RNA and control protein expression. Because these small RNAs utilize an intracellular RNA-silencing mechanism^9^, efficient target-RNA degradation can be achieved by simply expressing or introducing the small RNA, without overexpressing any exogenous protein. Because of their simple design and ease of use, siRNAs and miRNAs have been generalized as molecular tools for target-gene knockdown and applied for nucleic acid drug discovery^10^. RNA mutagenesis also has great potential as a versatile tool for biological, medical, and drug-discovery research. In contrast to RNA-interference technology, a general RNA-mutagenesis technology enabling RNA modification has not been established.

Adenosine-to-inosine (A-to-I) RNA editing, in which a specific adenosine is converted to inosine by an adenosine deaminase acting on RNA (ADAR), is a widely conserved post-transcriptional modification mechanism in metazoans^11^,^12^,^13^. A key role of A-to-I RNA editing is to recode genetic information at the transcript level, because inosine is read as a guanosine by the translation machinery. Hence, A-to-I RNA editing can potentially regulate various protein functions by changing target-protein codons.

Intracellular ribosomal RNA (rRNA) modifications, such as pseudouridylation^14^ and 2′-hydroxymethylation^15^, are processed by riboproteins composed of specific enzymes and small-nucleolar RNAs, which function as guide RNAs (gRNAs)^16^. The gRNA helps guide the protein enzyme towards the target by simple Watson–Crick base paring with its antisense sequence. Therefore, the modification activity can be redirected by changing the artificial antisense gRNA sequence^17^,^18^,^19^. Thus, gRNAs can effectively control target-RNA modification^20^.

Human ADAR2 (hADAR2) is mainly expressed in neurons^21^. hADAR2 is comprised of 2 double-stranded RNA-binding domains (dsRBDs) and a deaminase domain; thus, it preferentially targets adenosine located in double-stranded RNA (dsRNA), but not in single-stranded RNA (Fig. 1a). Because ADAR proteins are not riboproteins, they directly bind target RNAs via their own dsRBDs, without natural gRNA. Intelligent artificial editases were developed by tethering an artificial gRNA to modified deaminase domains from ADARs, using SNAP-tag technology^22^,^23^,^24^,^25^ or the well-known RNA-peptide-binding motif 26. These editases succeeded in regulating target-protein functions by site-directed A-to-I editing in a wide variety of cell types and in simple organisms, regardless of whether the natural ADAR protein was expressed. Instead, current site-directed RNA-editing strategies involve technical complexities, such as chemical modification and the functional requirement for unnatural deaminases. In the case of ADAR-expressing cells such as neurons, it should be possible to achieve an alternative site-directed RNA-editing strategy enabling direct editing of endogenous ADAR simply by providing the gRNA, without providing a modified deaminase. An effective way to develop this methodology would be to generate a novel gRNA-inducing natural ADAR protein to edit defined sites, and the development of such gRNA is currently under way^27^.

Here, we report ADAR-guiding RNA (AD-gRNA), which directly induces A-to-I mutations by guiding hADAR2 to the target-site (Fig. 1a). The editing activity of hADAR2 could be induced at a defined site by a programmable antisense sequence. In addition, site-directed RNA mutagenesis was achieved simply by transfecting hADAR2-expressing cells with a plasmid driving AD-gRNA expression. The AD-gRNA principle may serve as a foundation for a more generalized RNA-mutagenesis approach.

## Results

### Construction of hADAR2-guiding RNA for site-directed A-to-I RNA editing

**Figure 1:**
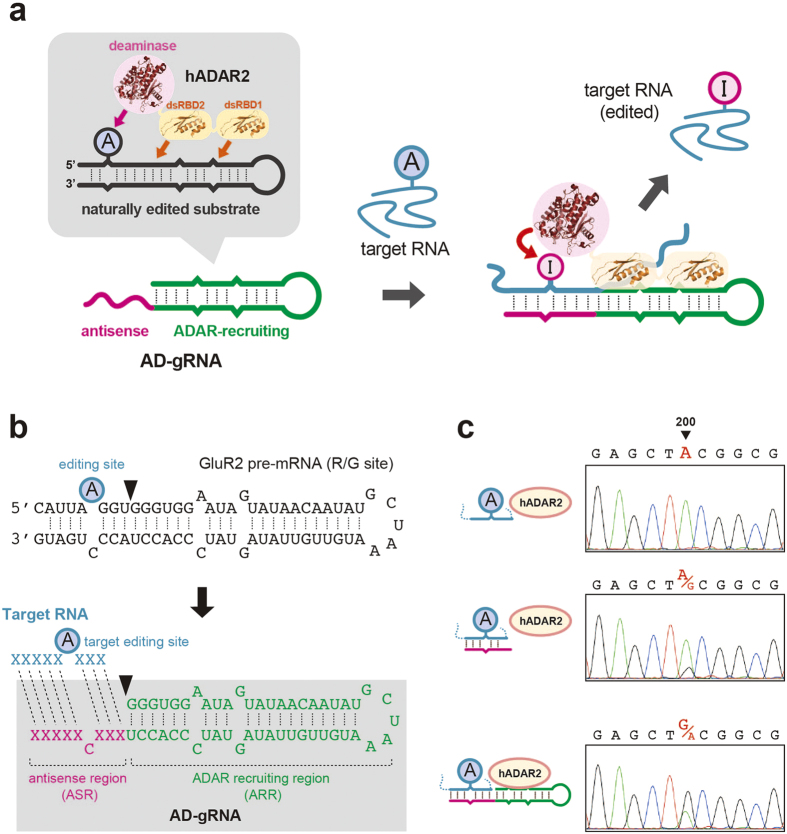
Construction of gRNAs inducing hADAR2 for site-directed A-to-I RNA editing. (**a**) Principle of the AD-gRNA strategy for site-directed RNA editing. Schematic representation of A-to-I RNA editing by hADAR2 is shown in the grey background. hADAR2 is composed of 2 dsRBDs and a deaminase domain. AD-gRNA, composed of an antisense region (magenta) and an ADAR-recruiting region (green) was designed based on a naturally edited substrate. A schematic representation of site-directed RNA-editing strategy using AD-gRNA is shown in the right panel. Target RNA is depicted with a turquoise line, and the black dashed line represents a base-pairing interaction. The target-editing site is marked by the circled A base. The AD-gRNA promotes both ADAR recruitment and target recognition by target-RNA hybridization. Thus, site-directed RNA editing is achieved by guiding hADAR2 onto the target site. (**b**) Sequence design of an AD-gRNA based on naturally edited substrate RNA. The well-known editing site in GluR2 RNA (R/G site) is indicated with the circled ‘A’. The position used to divide the GluR2 RNA into a prototype AD-guide RNA and a target-RNA is marked with a black arrowhead. The sequence of each fragment generated by the division is shown underneath. The AD-gRNA is shown within the grey background, and the antisense region (ASR) and ADAR-recruiting region (ARR) are shown in magenta and green, respectively. The target RNA is shown in turquoise characters. ‘X’ refers to any nucleotide, and the dotted lines connecting the blue and red characters indicate base pairing. (**c**) Editing-inducing activity of AD-gRNA. Sequencing chromatograms of the resultant sGFP cDNAs, which were obtained from RT-PCR followed by *in vitro* editing reaction using recombinant hADAR2 without gRNA (upper panel), with only the ASR (17 nt; middle panel), or with ADg-GFP_A200 (lower panel) are shown. Green and black peaks indicate signals for adenosine and guanosine, respectively. The target-editing site is indicated with a black arrowhead.

Our objectives were to develop a gRNA design to induce A-to-I RNA editing activity of native hADAR2 and assess its capacity for site-directed RNA mutagenesis. Essentially, gRNAs commonly have 2 modules: a protein-recruiting region and an antisense region that mediates target-RNA recognition through base paring. Importantly, proteins bound to gRNAs are positioned to effectively react with the target site after the antisense gRNA hybridizes to a target RNA. To construct human AD-gRNA with such functions, we focused on the secondary structure of the natural substrate RNA. dsRBDs mainly control substrate RNA binding, and the deaminase domain catalyses the hydrolytic deamination of adenosine (Fig. 1a). Accordingly, the substrate is thought to adapt a double-stranded structure required for dsRBD binding and enzymatic activity. We hypothesised that AD-gRNA could be designed from a natural substrate by utilising the dsRBD-binding region that contains the antisense sequence and forms base pairs around the edited site, so as to reconstitute the original substrate structure upon hybridization with a target RNA (Fig. 1a). We designed AD-gRNA based on the secondary structure of GluR2 (GRIA2) pre-mRNA, which is a well-characterized naturally edited hADAR2 substrate (Fig. 1b)^28^. The stem-loop structure of GluR2 RNA can be split into 2 components (Fig. 1b). One part of the stem-loop structure contains the target-editing site, and the other part contains the ADAR-recruiting region (ARR) and the antisense region (ASR) (Fig. 1b). The ASR was expected to determine the target-site by altering its sequence, based on the target-RNA sequence.

To validate the AD-gRNA design, we demonstrated AD-gRNA-induced RNA editing against a partial sequence of green fluorescent protein (GFP) mRNA from *Anthurium coerulescens*. A200 of GFP mRNA was the target adenosine, and a 160-nt short fragment of GFP mRNA (sGFP RNA) template (including A200) was constructed as the target RNA. AD-gRNA targeting A200 (ADg-GFP_A200) was designed with a 19-nt ASR and a 49-nt ARR (Fig. 1b; Supplementary Fig. 1), as described above. Both ADg-GFP_A200 and sGFP RNA were synthesised by *in vitro* transcription. Specific complex formation of ADg-GFP_A200 with sGFP RNA (depending on the antisense sequence) was confirmed in gel-mobility shift assays, followed by an annealing reaction (Supplementary Fig. 2). After complex formation, purified recombinant hADAR2 (Supplementary Fig. 3) was added to the annealed gRNA-target RNA complex and incubated for 1 h for the *in vitro* editing reaction. The editing efficiency at A200 on sGFP RNA was analysed by the fluorescent dye-terminator sequencing method^29^, followed by reverse transcription (RT) and the polymerase chain reaction (PCR) (Fig. 1c). Editing was not detected by sequencing in the absence of AD-gRNA, including A200 (Fig. 1c and Supplementary Fig. 4). With ASR alone (19-nucleotide [nt] antisense RNA including 1 mismatched base deleted from the AD-gRNA ARR), slight editing (21%) was detected at A200, as determined by estimating the peak-height ratio for G/A (Fig. 1c). In contrast, significant editing (66%) was observed with ADg-GFP_A200 designed to fully match the AD-gRNA. The improved editing efficiency indicated that the ARR could induce efficient editing. We also analysed editing at all adenosines in the obtained sequencing chromatogram of sGFP cDNA to assess the off-target editing induced by the AD-gRNA (Supplementary Fig. 4). No distinct off-target editing was detected under these reaction conditions.

**Figure 2:**
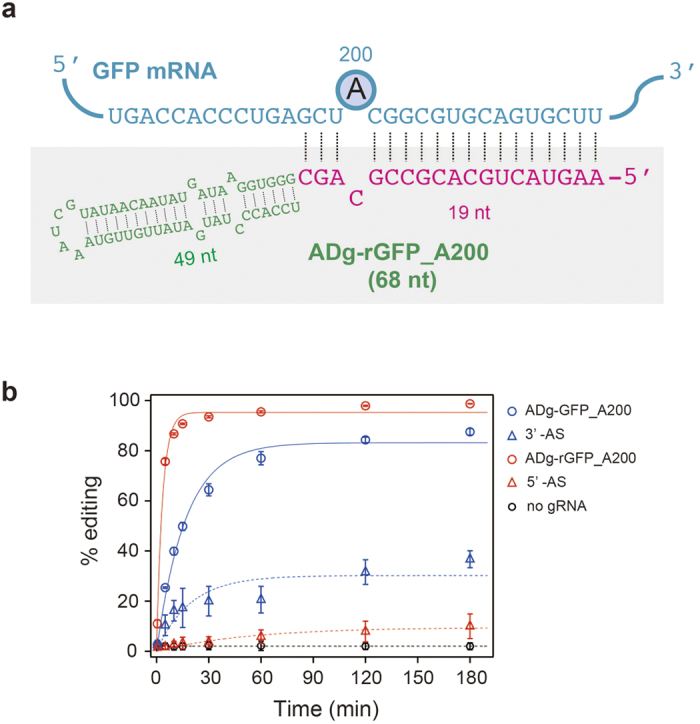
Design and editing induction activity of 5′-AS AD-gRNA. (**a**) Nucleotide sequence of ADg-rGFP_A200 and a partial sequence of sGFP RNA are shown as a complex with a predicted secondary structure. sGFP RNA is represented in blue, and the target-editing site (A200) is depicted using a circled ‘A’. ASR and ARR are shown in red and green characters, respectively. (**b**) *In vitro*-editing induction activity of AD-gRNA. Changes in the editing ratio at A200 over time with 3′-AS AD-gRNA (ADg-GFP_A200, blue open circles), 3′-AS alone (blue open triangles), 5′-AS AD-gRNA (ADg-rGFP_A200, red open circles), 5′-AS (red open triangle), or without gRNA (black open circles). Each editing percentage was quantified by measuring the peak heights for A and G generated from the direct-sequencing chromatograms (Supplementary Figs 4 and 5) and calculated as follows: (height of the G peak)/(height of A peak + height of G peak). The results are presented as averages with standard deviations from 3 independent experiments.

We next extended the diversity of functional designs for AD-gRNA. Based on the AD-gRNA design described above, the antisense region should be positioned at the 3′-end of the ARR. A *trans*-type substrate structure, composed of the gRNA and target RNA, would not involve a phosphodiester bond between the strand containing a target-site and the ARR. Hence, the relative distance and direction of the ARR from the target-site should be flexible. We hypothesized that AD-gRNA possessing an ASR at the 5′-end of the ARR could induce a cognate editing substrate structure when the ASR hybridizes to the target RNA. To demonstrate the utility of this 5′-AS AD-gRNA design, ADg-rGFP_A200 was generated by introducing the ASR into the 5′-end of the ARR to target A200 in sGFP RNA (Fig. 2a). ADg-rGFP_A200 showed specific activity against A200 (Fig. 2b and Supplementary Fig. 5). Interestingly, the editing efficiency of 5′-AS AD-gRNA exceeded that of 3′-AS AD-gRNAs (Fig. 2b). Off-target editing was not detected with sGFP RNA and ADg-GFP_A200 (Supplementary Fig. 5). Consequently, we succeeded in developing a highly active AD-gRNA framework by introducing the antisense region into the 5′-end of the ARR.

We also tested the utility of other AD-gRNA structural frameworks. A 40-nt hairpin structure serving as an hADAR2 substrate was previously designed by modifying the hairpin region on GluR2 RNA^30^,^31^ (Supplementary Fig. 6A). We expected that it would be possible to construct a shorter AD-gRNA (sAD-gRNA) if the AD-gRNA design were applied to the hairpin substrate. To test the sAD-gRNA design, sADg-GFP_A200 and sADg-rGFP_A200 were constructed based on the hairpin substrate used for the 5′-AS and 3′-AS AD-gRNA designs (Supplementary Fig. 6B). After the sGFP RNA-editing reaction, A200 was specifically edited, depending on the sAD-gRNA used, with editing efficiencies of 66% and 95% by sADg-GFP_A200 and sADg-rGFP_A200, respectively (Supplementary Fig. 6C). Off-target editing was not detected in either reaction (Supplementary Fig. 7).

Next, we performed editing assays using full-length GFP mRNA (720 nt). Both ADg-GFP_A200 and ADg-rGFP_A200 efficiently induced A-to-I editing in A200 (Supplementary Fig. 8). In the sequencing chromatograms, distinct off-target editing (>5%) was detected at several sites (Supplementary Fig. 9). In the editing reaction without gRNA, sites 125 (7%), 385 (14%), 407 (6%), 470 (10%), and 500 (52%) (numbers in parentheses indicate the calculated editing percentages at each site) were edited by hADAR2 in a gRNA-independent manner. Although off-site editing still occurred in the editing reaction with AD-gRNA, the editing efficiencies were changed at some sites. For instance, the editing percentage was increased to 12–16% at site 125 and was significantly decreased to 14–24% at site 500. These results showed that detectable off-target editing in this analysis was highly dependent on the target RNA, but was not strongly induced by AD-gRNA.

The AD-gRNA reported here was constructed using a basic framework, wherein the dividing line between both RNA components was fixed 3 nt downstream from the target-editing site (Fig. 1b). The editing efficiency decreased when this dividing line was 0 or 1 nt, but nearly constant editing activity was observed at distances of 2–5 nt away (Supplementary Fig. 10). Therefore, we used the 3-bp framework in this study.

### dsRBD-dependence of AD-gRNA-induced site-directed RNA editing

**Figure 3:**
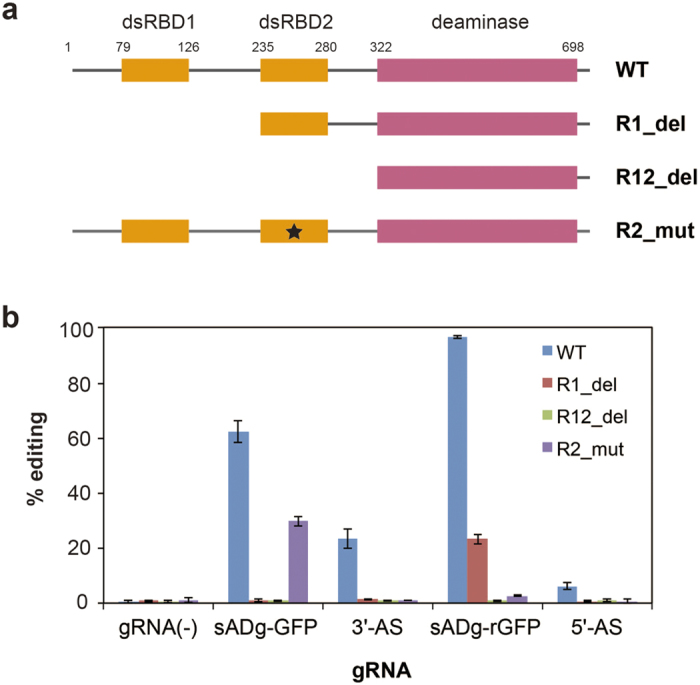
Analysis of dsRBD-dependent, AD-gRNA-induced site-directed RNA editing. (**a**) Schematic representation of the hADAR2 mutants used in this study. The regions corresponding to the dsRBDs and deaminase domain are represented in orange and magenta. The numbers denote the amino acid positions, relative to the N terminus of hADAR2. The black star indicates the mutation causing a defect in dsRNA binding by the dsRBD. (**b**) Editing percentages at A200 in sGFP RNA during the AD-gRNA editing-induction reaction, with each hADR2 mutant. Using wild-type hADAR2 (blue), R1_del (red), R12_del (green) and R2_mut (purple), editing reactions were performed without gRNA (gRNA[-]), with sADg-GFP_A200 (ADg-GFP) and its ASR (3′-AS), and with sADg-rGFP_A200 (ADg-rGFP) and its ASR (5′-AS). The editing percentages shown were calculated from the peak heights for A and G generated from the direct-sequencing chromatograms, as follows: (height of the G peak)/(height of A peak + height of G peak). The results are presented as averages with standard deviations from 3 independent experiments.

To clarify the dsRBD-dependence of the ARR on AD-gRNA, we performed editing assays using dsRBD-deleted or -mutated hADAR2 (Fig. 3a). We prepared deletion mutants with dsRBD1 and/or dsRBD2 deleted from wild-type hADAR2 (Supplementary Fig. 3). The editing efficiency at A200 on sGFP RNA was analysed after the editing reaction with AD-gRNAs and ADAR mutants. With the dsRBD1 deletion mutant (R1_del), significant reduction of editing efficiency was observed after AD-gRNA-induced editing (Fig. 3b), and the editing signal on 3′-AS AD-gRNA (sADg-GFP) almost disappeared. Although 5′-AS AD-gRNA (sADg-rGFP) retained small editing-induction activity, this editing did not in reactions with the dsRBD1- and dsRBD2-deletion mutant (R12_del), containing the deaminase domain only. Next, we determined which dsRBDs contribute more to efficient AD-gRNA-dependent editing induction. Because the deletion mutant could not be used to assess the effect of dsRBD2 alone, another ADAR2 mutant, deficient in the dsRNA-binding activity of dsRBD2, was constructed by introducing amino acid mutations into dsRBD2 (R2_mut). The editing efficiency actually decreased comparing to that observed with wild-type hADAR2.

### Regulation of functional protein expression using AD-gRNA-induced A-to-I editing *in vitro*

**Figure 4:**
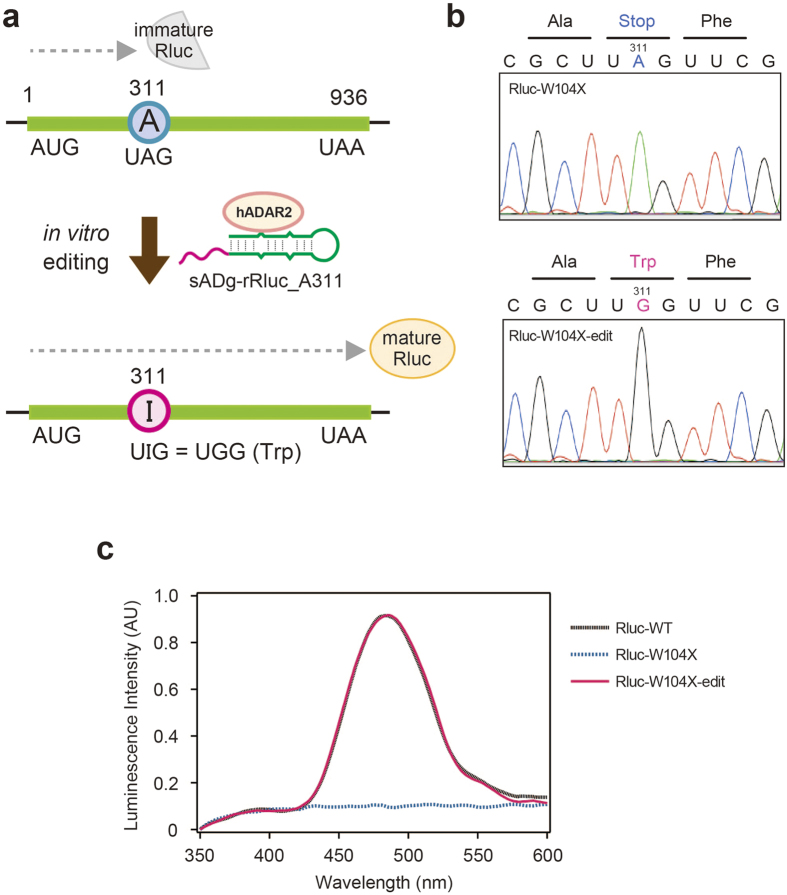
Amber codon-repair experiment by AD-gRNA-induced RNA editing. (**a**) Schematic representation of a codon-repair experiment conducted using a modified luciferase reporter. The modified Renilla luciferase mRNA (Rluc-W104X) was generated by changing guanosine at nucleotide 311 to adenosine (A311) to alter codon Trp104 (UGG) into an amber stop codon (UAG). Active mature luciferase was translated from Rluc-W104X after A311 was edited to I311 by ADg-rRluc_A311. (**b**) Sequence chromatograms of cDNA from Rluc-W104X (upper) and *in vitro-*edited Rluc-W104X with ADg-rRluc_A311 (Lower). (**c**) Confirmation of active luciferase expression regulated by AD-gRNA. Luminescence-spectrum analysis of samples after performing an *in vitro* translation reaction with wild-type luciferase mRNA (Rluc-WT, black), Rluc-W104X (blue), or *in vitro* edited-Rluc-W104X (magenta).

As inosine is read as guanosine by the translation machinery, a promising application of site-directed RNA mutagenesis is to regulate the expression or function of target proteins by introducing specific codon changes. Previous site-directed RNA-editing strategies have achieved specific codon changes to regulate target-gene functions, for example, Stop (UAG) to Trp (UGG), Tyr (UAC) to Cys (UGC), and Ser (AGC) to Gly (GGC) in the open-reading frame of enhanced-fluorescent proteins^24^, or the cystic fibrosis transmembrane-conductance regulator^26^. Indeed, codon-repair activities have been as a reporter in cultured cells^24^ and in living organisms^32^. Thus, we performed an amber codon-repair experiment to demonstrate the feasibility of such experiments using our AD-gRNA. We constructed a novel *in vitro* assay system for this study, in which the *in vitro*-editing and *in vitro*-translation reactions were performed sequentially, without using a cellular system. A modified Renilla luciferase mRNA (Rluc-W104X), in which G311 was changed to A311 to alter Trp104 (UGG) into an amber stop codon (UAG), was used as a reporter (Fig. 4). Active, mature luciferase could be translated after A311 was edited to inosine, regenerating the Trp codon (UIG; Fig. 3a). As an AD-gRNA, sADg-rRluc_A311 was constructed to repair the amber codon on Rluc-W104X (Supplementary Fig. 11). After the *in vitro*-editing reaction with sADg-rRluc_A311, the editing efficiency at A311 and off-target editing were analysed in sequencing chromatograms (Supplementary Fig. 12). The A311 target site was almost completely edited in the presence of sADg-rRluc_A311 (Fig. 3b), indicating that AD-gRNA could be used for codon alteration and regulating functional protein expression. Although Rluc-W104X RNA itself was subject to editing at A122 (~80%) and A215 (~15%), ADg-RNA dependent off-target editing was not detectable (Supplementary Fig. 12).

Next, a luciferase assay was performed following *in vitro* translation, using the edited Rluc-W104X template (Fig. 4c), to study active luciferase expression triggered by the AD-gRNA induced-codon change. Active luciferase expression was not observed following translation of the unedited Rluc-W104X transcript. However, luminescence was detected after translating the edited Rluc-W104X transcript. In addition, the recovered luminescence reached an intensity obtained using wild-type Rluc mRNA (Rluc-WT).

### Intracellular site-directed RNA editing by AD-gRNA in ADAR-expressing cells

**Figure 5:**
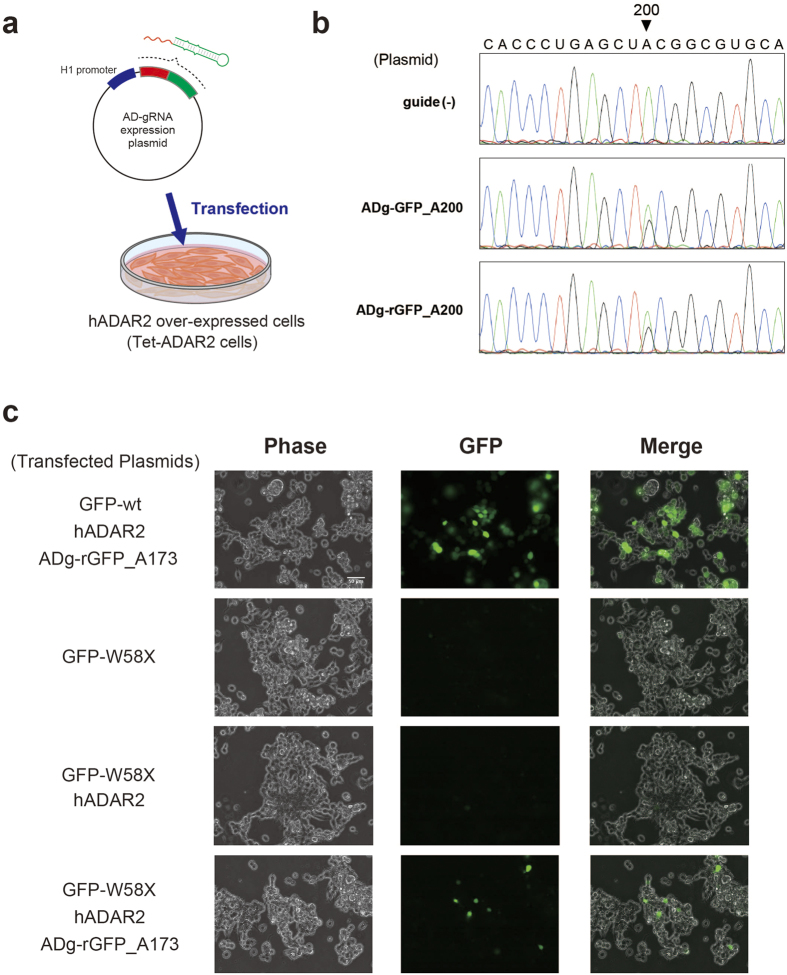
Application of AD-gRNA for site-directed RNA mutagenesis and regulating target-protein expression in cells. (**a**) Site-directed RNA mutagenesis with AD-gRNA following plasmid transfection in hADAR2-over-expressed cells. The expression plasmid for AD-gRNA was constructed using a pol III-driven short RNA expression vector. Previously constructed tet-ADAR2 cells, in which both hADAR2 and AcGFP expression can be controlled under a Dox-inducible promoter, were used as hADAR2-over-expressed cells. (**b**) Confirmation of specific editing-induction activity of ADg-GFP_A200 and ADg-rGFP_A200 in tet-ADAR2 cells. Sequencing chromatograms of GFP cDNA obtained from cells cultured without plasmid transfection (guide [-], upper), with p-ADg-GFP_A200 (middle), or p-ADg-rGFP_A200 (lower) are shown. The target adenosine (A200) is indicated with a black arrowhead. All sequencing chromatograms are shown in Supplementary Fig. 18. (**c**) Fluorescent microscopy pictures in the intracellular codon-repair experiment. GFP-W58X is a fluorescent reporter for real-time monitoring of functional protein expression by amber codon repair induced by AD-gRNA. The plasmids transfected are shown on the left of the pictures. The top pictures show results from control experiments, in which HEK293 cells were co-transfected with a wild-type GFP expression plasmid (GFP-wt), p-hADAR2, and p-ADg-rGFP_A173. The lower pictures show the cells co-transfected with the reporter plasmid (GFP-W58X), p-hADAR2, and p-ADg-rGFP_A173. Fluorescent micrographs for each cell were obtained at 48 h post-transfection.

The most significant characteristic of AD-gRNA is its ability to induce editing of native hADAR2. If target cells express sufficient hADAR2 to induce editing, then intracellular RNA mutagenesis could be achieved by simply introducing AD-gRNA. Firstly, we checked the intracellular editing-induction activity of ADg-GFP_A200 and ADg-rGFP_A200, which were already characterized in the above *in vitro* experiments. We used the previously established Tet-ADAR2 cell line^33^, in which hADAR2 and AcGFP could be simultaneously over-expressed under the control of a doxycycline (Dox)-inducible promoter (Fig. 5a). Cells were transfected with pSUPER.neo plasmids encoding AD-gRNAs. The pSUPER.neo plasmid is commonly used for pol III-driven expression of short target RNAs (Fig. 5a). After plasmid transfection and culturing with Dox, hADAR2 and AD-gRNA expression in Tet-ADAR2 cells were checked by western blotting and real-time PCR, respectively (Supplementary Fig. 13 and 14). To confirm the feasibility of intracellular editing, we analysed the editing efficiency at a Q/R site in filamin A (FLNA)^34^ mRNA and a Y/C site in Blcap bladder cancer-associated protein (BLCAP)^35^ mRNA, which are well-known editing sites for hADAR2 and hADAR1, respectively (Supplementary Fig. 15). After Tet-ADAR2 cells were cultured with 5 μg/ml Dox, the editing efficiency on FLNA increased significantly, but little BLCAP mRNA was observed (Supplementary Fig. 15).

To analyze intracellular editing induced by AD-gRNA, we next determined the editing efficiency at A200 in GFP mRNA from Tet-ADAR2 cells transfected with AD-gRNA-expression plasmids (p-AD-gRNA). In the sequencing chromatograms of GFP mRNA obtained from p-AD-gRNA-transfected cells, editing of the A200 target site depended on AD-gRNA expression (Fig. 5b). The editing efficiencies at A200 in cells expressing the ADg-GFP_A200 and ADg-rGFP_A200 transcripts were 32% and 28%, respectively (Fig. 5 and Supplementary Fig. 16), showed that the editing-induction efficiency of ADg-GFP_A200 was slightly higher than that of ADg-rGFP_A200. Interestingly, this trend did not correspond to that observed in the *in vitro* experiment (Fig. 2b). After transfecting the expression plasmid encoding the 5′-AS region alone (p-5′-AS), editing was not detectable in the cells (Supplementary Fig. 16), indicating that the ARR functioned efficiently in site-directed RNA editing in cells. In addition, sADg-GFP_A200 and sADg-rGFP_A200 (shorter AD-gRNAs) also induced intracellular editing, with editing percentages of 24% and 25%, respectively (Supplementary Fig. 16). The editing efficiency of short ADg-RNA was slightly lower than those of GluR2-based AD-gRNAs.

We next verified the frequency of off-target intracellular editing on AcGFP mRNA induced by AD-gRNA expression. Analysis of all adenosines in the obtained sequencing chromatograms showed that distinct off-target editing did not occur at any adenosine in the GFP sequence (Supplementary Fig. 17).

Because AD-gRNA can induce wild-type hADAR2, it potentially interfered with intracellular editing homeostasis. We analysed the effect of AD-gRNA expression on intracellular editing in Tet-ADAR2 cells by monitoring changes in the editing efficiencies with an endogenous editing substrate after p-AD-gRNA transfection. Thus, we determined the editing efficiencies at the Q/R site in FLNA and the Y/C site in BLCAP mRNA (Supplementary Fig. 18). With both mRNAs, distinct differences in the editing efficiency were not observed between cells transfected with or without p-AD-gRNA (Supplementary Fig. 18).

Finally, we performed an intracellular codon-repair experiment to demonstrate the potential of AD-gRNA in regulating target-protein expression. To visualize intracellular functional-protein expression caused by AD-gRNA-induced codon repair, GFP-W58X mRNA^26^,^32^ was used as a reporter gene. ADg-rGFP_A173 was designed to convert the stop codon to a Trp codon, using the pSUPER.neo vector. All components needed for this intracellular codon-repair experiment (including AD-gRNA, hADAR2, and the reporter) were over-expressed in HEK293 cells by plasmid co-transfection, as the Tet-ADAR2 cells described above co-expressed GFP and hADAR2 after Dox-dependent induction. The p-hADAR2 and p-GFP-W58X plasmids were generated using the pcDNA3.1 vector, which is commonly used for pol II-driven protein expression. In addition, a wild-type GFP expression plasmid (p-wtGFP) was also constructed using the same vector for control experiments. These expression plasmids were co-transfected into HEK293 cells using a variety of combinations. At 48 h post-transfection, GFP expression in each group of plasmid-transfected cells was observed by fluorescence microscopy (Fig. 5c). Fluorescent cells were readily observed in p-wtGFP-transfected control cells. Under these experimental conditions, 101 cells with distinct fluorescent were identified in ~280 observable cells in the representative image. Positive cells showing strong fluorescence were not identified in cells transfected with p-GFP-W58X. Co-transfection of p-hADAR2 and p-GFP-W58X also did not generate positive fluorescent cells. In contrast, fluorescent cells were clearly observed after co-transfection with the p-ADg-rGFP_A173, p-GFP-W58X, and p-hADAR2 expression vectors. Approximately 10 fluorescent cells were observed out of 290 cells total (Fig. 5c). Moreover, fluorescent cells were not generated following 5′-AS transfection. These results clearly showed that ADg-rGFP_A173 regulate mature GFP biosynthesis by inducing site-directed A-to-I RNA editing.

## Discussion

A versatile method for targeting intracellular A-to-I RNA editing would be a significant breakthrough toward the establishment of practical site-directed RNA mutagenesis. Previously, artificial gRNAs were used to direct the RNA-modification activities of natural riboproteins^17^,^18^,^19^. However, gRNAs for ADARs have not been discovered in nature. Here, we contrived gRNA-based, site-directed RNA editing utilizing an endogenous RNA-editing mechanism by developing a novel gRNA that can freely induce A-to-I RNA editing at a programmable site, via the native hADAR2 protein.

AD-gRNAs could be simply designed, based on the secondary structure of natural or artificial editing substrates containing the editing site for hADAR2. In the case of GluR2 RNA, the binding region for hADAR2 dsRBDs was solved by solution-structure analysis^36^. Considering this structural information, GluR2 RNA could be divided conceptually into one segment containing the target-RNA containing editing site and another segment composed of the ARR and ASR, which served as a prototype AD-gRNA (Fig. 1b). The cognate structure serving as the editing substrate was expected to reconstitute by base pairing between the target RNA and the antisense region. With this design, target adenosine can be defined by modifying the antisense region to match the target sequence. Most recently, it was reported that artificial gRNA could be constructed based on a similar, but not identical design concept, relative to our study^27^. Indeed, our AD-gRNA also achieved site-directed RNA editing at the site programed in the ASR using this design concept (Fig. 2 and 4). AD-gRNA-induced RNA editing is principally dependent upon the intrinsic properties of hADAR2. Hence, the efficiency of editing target adenosines was expected to be highly dependent upon its neighbouring sequences^37^. Thus, adenosines that can be targeted by our AD-gRNA system will be restricted to some extent by the neighbour preferences of hADAR2. In addition to the AD-gRNA design discussed above (3′-AS AD-gRNA design, in which the ASR is positioned at the ARR 3′-end), we succeeded in developing another AD-gRNA framework (5′-AS AD-gRNA design, in which the ASR is positioned at the ARR 5′-end). Based on the 3′-AS AD-gRNA design (Fig. 1b), the original editing site with the 5′-AS AD-gRNA design (Fig. 2a) should be placed essentially in the position of the cytosine-forming mismatched nucleotide with the target adenosine. Though the design concept was distinct from 3′-AS AD-gRNA design, 5′-AS AD-gRNA indeed showed specific editing-induction activity (Fig. 2b), suggesting that such editing occurred via a mechanism that differed from that of the 3′-AS AD-gRNA. This expectation was also supported by different dsRBD dependences, in which 5′-AS AD-gRNA showed a higher dsRBD2 dependence, but 3′-AS AD-gRNA was dependent upon dsRBD1 (Fig. 3). Thus, different editing-induction mechanisms might exist between 5′-AS AD-gRNA and 3′-AS AD-gRNA.

Because hADAR2 preferentially edits adenosines in dsRNA, simple antisense RNA can potentially serve as a guide RNA. Indeed, the antisense region of ADg-GFP_A200 induced A-to-I editing (Fig. 1c). However, the efficiency was significantly lower than that observed with the complete AD-gRNA, both *in vitro* and in cells. These results clearly showed that the ARR of our AD-gRNAs promoted efficient editing. Although the editing efficiency was relatively lower, dsRBD-dependent editing was also observed with the ASR alone, which clearly indicated that antisense region-induced editing was not responsible for only deaminase domain. In addition, the efficiency of ASR-induced editing appeared highly dependent on the target sequence (Fig. 2b). Although these observations suggest the possibility of site-directed RNA editing using simple antisense RNA, it is clear that AD-gRNA was more effective.

We found that the AD-gRNA showed the potential for off-target editing by ADAR.

*In vitro*-editing analysis in the structured region of GluR2 RNA showed a low propensity for off-target editing promoted by the ARR (Supplementary Fig. 19). With the ASR, off-target editing would also be expected to depend on the length and sequence involved. Undesirable editing by ADAR can be efficiently suppressed by introducing a mismatched nt into the complementary position against the adenosine of interest or by modifying the neighbouring nt in the antisense region^23^. It should be possible to apply this design strategy with AD-gRNAs to enhance target selectivity. To achieve efficient and specific editing, the ASR length and sequence should be tuned according to the target-RNA sequence.

Successful modification of the ARR strongly indicated the potential for constructing AD-gRNAs against other ARRs from naturally edited substrates, such as the GluR2 Q/R^38^ and NEIL1 K/R sites^39^. We did not perform editing assays using hADAR1, which is another cellular editing enzyme. Because hADAR1 has a similar domain structure compared to that of hADAR2^12^,^40^, our design strategy is expected to apply to both hADAR2- and hADAR1-guiding RNAs.

Functional tuning of gRNA for target selectivity and efficiency are considered important requirements for generally applicable mutagenesis methods. In our AD-gRNA design, the dividing line separating GluR2 RNA into target-RNA and AD-gRNA (Supplementary Fig. 12) can potentially be utilized to generate further basic AD-gRNA frameworks to diversify the gRNA function.

Intracellular site-directed RNA editing was achieved simply by expressing AD-gRNA in hADAR2-over-expressing cells. In addition, our codon-repair experiments (both *in vitro* and in cells) clearly showed that AD-gRNA could be used to change codons, thereby altering protein expression. In this intracellular codon-repair experiment, the efficiency of AD-gRNA-induced GFP expression was low (Fig. 5c), which was attributed to an inefficiency of editing induction under the same conditions used for fluorescence microscopy-based observations (Supplementary Figs 20 and 21). Actually, the editing efficiency of the endogenous hADAR2-editing substrate, FLNA RNA, was also lower than that in tet-ADAR2 cells (Supplementary Fig. 20B), indicating that the editing state in plasmid-transfected cells were lower than that in tet-ADAR2 cells. Accordingly, this editing state may have been a main reason for the inefficiency of editing induction, resulting in a small number of fluorescent cells. Thus, the intracellular editing state in target cells appears to be a critical factor influencing efficient AD-gRNA-induced site-directed editing. In addition, the AD-gRNA can potentially achieve site-directed A-to-I RNA editing simply by its introduction or expression when the target cell is in an intrinsically high editing state.

With our method, the intracellular editing-induction efficiency could be highly affected by many factors, including the expression and intracellular stabilities of hADAR2 and AD-gRNA. In addition, intracellular localization might also be key for efficient editing induction because hADAR2 is localized in the nuclear compartment, especially in the nucleolus^41^. Therefore, the *in vitro* capability of AD-gRNA may not be directly reflected in cells. Editing activities observed *in vitro* (for example, sADg-rGFP_A200 was more active than sADg-GFP_A200), were not perfectly replicated in cells. Additional studies are needed to understand such differences in activity.

RNA editing serves a critical role in cellular homeostasis. Hence, deregulation of ADAR activities caused by mutation or changes in its expression level have been related to wide variety of human diseases^42^,^43^, including neurodevelopmental disorders^44^,^45^, viral infection and auto-immune disorders^46^, and cancer^47^,^48^. ADAR-dependent RNA mutagenesis can potentially affect RNA-editing homeostasis, regardless of whether modified ADAR or natural ADAR is used. Although the inhibitory effect of AD-gRNA on the endogenous editing state could be undetectably small at least during ADAR2-overexpression (Supplementary Fig. 15), the effect on the original editing state by these RNA-mutagenesis methods will have important implications for practical usage.

A-to-I RNA editing can potentially regulate various critical protein functions within cells, such as enzyme catalysis and signal transduction, by changing the codon^20^. Therefore, our AD-gRNA strategy has great potential as a basic molecule for establishing a versatile site-directed RNA mutagenesis method to regulate a target-protein functions in ADAR2-expressing cells.

In summary, we developed a novel gRNA that can induce A-to-I mutations by guiding hADAR2 to the target site. AD-gRNA specifically introduced A-to-I mutations into target sites that were programed into the antisense region. Moreover, site-directed RNA editing could be achieved by only introducing gRNA into cells that over-expressed hADAR2. Our gRNA strategy has the potential to provide a basic framework for establishing a generally applicable RNA-mutagenesis approach.

## Methods

### Oligonucleotides

DNA oligonucleotides and synthetic RNAs were purchased from Hokkaido System Science Co., Ltd. (Hokkaido, Japan) and Sigma Genosys (Hokkaido, Japan). The sequences of all DNA oligonucleotides and synthetic RNAs used in this study are listed in Supplementary Table 1.

### Preparation of AD-gRNAs and target RNAs

AD-gRNAs were synthesized by *in vitro* transcription. First, template dsDNA for *in vitro* transcription was synthesized using synthetic oligonucleotides. In general, 1 μM of the forward DNA oligonucleotide containing the T7 promoter sequence and 1 μM of a reverse DNA oligonucleotide were mixed in annealing buffer (50 mM Tris-HCl [pH 7.6] and 50 mM NaCl), after which the annealing reaction was performed by denaturing the mixture at 95 °C for 3 min, followed by cooling to room temperature for 15 min. To generate dsDNA templates, the annealing product was elongated using Klenow polymerase (New England BioLabs). The obtained dsDNA fragment was purified by phenol/chloroform extraction and ethanol precipitation. Using purified dsDNA as a template, *in vitro* transcription reactions were performed using the AmpliScribe T7 Kit (Epicentre Biotechnologies), according to the manufacturer’s protocol. Reaction solutions were subjected to phenol/chloroform extraction and ethanol precipitation, and finally AD-gRNAs were purified from a denaturing 8% polyacrylamide gel containing 8 M urea. In the case of target RNAs, dsDNA templates were initially prepared by PCR, using standard PCR conditions (PrimeSTAR GXL DNA polymerase, Takara Bio). Subsequently, *in vitro* transcription and purification were performed as described above. The sequences of all DNA oligonucleotide and RNA samples used in this study are shown in Supplementary Table 1.

### Preparation of recombinant hADAR2

Recombinant hADAR2 was prepared as described previously. Briefly, the coding region for human ADAR2 was PCR-amplified from a cDNA clone (clone ID 6014605, Open Biosystems) and then cloned into the yeast expression vector pYES2/NT A (Invitrogen) that expresses N-terminally His-Express-tagged fusion proteins. The obtained plasmid was transformed into INVSc1 yeast cells (Invitrogen) using the Frozen-EZ Yeast Transformation II Kit (Zymo Research). Transformants were cultured in liquid medium, and recombinant hADAR2 was purified using a HisTrap HP column (GE Healthcare). Fractions containing hADAR2 were collected and dialyzed against storage buffer (10 mM Tris-HCl [pH 7.5], 150 mM NaCl, 5% glycerol, 1 mM DTT) using a 50-kDa molecular weight cut-off Float-A-Lyzer G2 (Spectra/Por). Purified hADAR2 was quantified using the DC Protein Assay Kit (BioRad), according to the manufacturer’s instructions.

To prepare dsRBD-binding defective hADAR2 mutants, dsRBD-deleted and -mutated hADAR2 mutants were designed with references to previous reports^49^,^50^. In the case of the dsRBD-deleted hADA2 mutants, we designed R1_del (hADAR2 amino acids 232–701) as the dsRBD1-deleted hADAR2 by truncating the wild-type hADAR amino acid sequence from the N-terminus to the end of dsRBD1. For the deletion mutant lacking both dsRBD1 and dsRBD2, R12_del (hADAR2 amino acids 317–701) was generated. In dsRBD2-mutated hADAR2 variants (in which binding affinity of dsRBD2 was defective), K127, K128, and K131 were mutated into E127, A128, and A131, respectively. The cloned DNA inserts from the deletion mutants were amplified by PCR using specific primers (ADAR2_R1del_F and ADAR2_ScEx_XbaR for R1_del; ADAR2D_ScEx_F and ADAR2_ScEx_XbaR for R12_del) and then cloned into the same yeast expression vector. To construct the cloning insert DNA for R2_mut, respective point mutations were introduced into wild-type hADAR2 by PCR using the ADAR2_forR2mut_F and ADAR2_fotR2mut_R primers, with the QuikChange Site-Directed Mutagenesis Kit (Stratagene). The constructed expression plasmids were transformed into INVSc1 yeast cells. Purification and quantification for each hADAR2 mutant were performed as described above.

### *In vitro* editing assay

To generate gRNAs and target-RNA complexes, 900 nM AD-gRNA and 300 nM target-RNA were annealed by heating at 80 °C for 3 min with subsequent slow cooling to 25 °C at 1 °C/10 s in annealing buffer (10 mM Tris-HCl [pH 7.6] and 150 mM NaCl). The editing reaction was performed as follows: 5 nM complex and various concentrations of purified hADAR2 were mixed in 20 μL of reaction buffer (20 mM HEPES-KOH [pH 7.5], 100 mM NaCl, 2 mM MgCl_2_, 0.5 mM DTT, 0.01% TritonX-100, 5% glycerol, 1 U/μL Murine RNase Inhibitor (New England BioLabs) and then incubated at 37 °C for 2 h. Subsequently, the reacted RNA was purified by phenol/chloroform extraction and ethanol precipitation, after which the RNA pellet was dissolved in 5 μL TE buffer. To generate cDNA from the reacted RNA, the purified RNA was reverse transcribed using the PrimeScript II Reverse Transcription Kit (Takara Bio). cDNA was PCR-amplified using PrimeSTAR GXL DNA polymerase (Takara Bio), following a standard protocol. The editing efficiency at each site was analysed by direct sequencing, as follows. Ten nanograms of gel-purified PCR product was sequenced using a reverse primer and the BigDye Terminator Cycle Sequencing Kit (Applied Biosystems). Next, sequence chromatograms were obtained using a 3500 Genetic Analyzer (Applied Biosystems). The editing ratio at each site was calculated using the following equation: A/[A + G], where A and G correspond to adenosine and guanosine peak heights measured using Sequence Scanner software ver. 1.0 (Applied Biosystems), respectively. The fitting curves were calculated using the single-exponential equation: F_t_ = F_0_ + F_1_ (1 − e^−*k*t^), where F_0_ and F_1_ represent the editing fraction at time 0 and at the reaction end point, respectively, and where k is the first-order editing rate constant.

### *In vitro* codon-repair experiments using a luciferase reporter assay coupled with *in vitro* translation

The reporter for the *in vitro* codon-repair experiment was designed based on Renilla luciferase mRNA (Rluc-WT). The reporter mRNA, Rluc-W104X, was prepared by mutating G311 to A311 in codon Trp104 (UGG) in Rluc-WT, using the QuikChange Site-Directed Mutagenesis Kit (Stratagene). With this Rluc-W104X reporter, the full-length, wild-type Rluc transcript was synthesized when A311 was edited to I311. Sequential *in vitro* editing and translation reactions were performed as follows. First, 5 μM Rluc-W104X and 15 μM sADg-rRluc_A311 were annealed by heating them together at 80 °C for 3 min, with subsequent slow cooling to 25 °C at 1 °C/10 s in annealing buffer. Then, 0.5 μM of the reporter-gRNA complex was subjected to an editing reaction with 1.25 μM hADAR2 in the reaction buffer at 37 °C for 2 h. In parallel, a control sample (lacking gRNA) was also tested using the same process. The edited RNA sample was recovered by phenol/chloroform extraction and ethanol precipitation. To assess whether active Rluc could be translated from the edited reporter mRNA, an *in vitro* translation reaction was performed in 20 μl rabbit reticulocyte lysate (Promega), using 1 μg of reporter RNA obtained from the editing reaction. After translation at 37 °C for 1 h, a chemiluminescence reaction was performed using the Dual-Luciferase Reporter Assay System (Promega), according to the manufacturer’s protocol, and luminescence was measured with a spectrophotometer.

### Preparation of the AD-gRNA expression plasmid

An AD-gRNA expression plasmid was constructed using pSUPER.neo (Oligoengine), which is used for expressing short cellular RNAs such as short hairpin RNAs and miRNAs, based on the H1 RNA polymerase III promoter. While preparing DNA inserts encoding AD-gRNAs, tetra uridine sequences were inserted into the 3′-end of the AD-gRNA sequence to terminate pol III transcription at a defined position. Cloning into the vector was facilitated by introducing *Bgl* II and *Hind* III restriction sites into the 5′ and 3′ ends, respectively. The DNA inserts were generated using synthetic oligonucleotides, which are shown in Supplementary Table 1. Each DNA insert was cloned into the pSUPER.neo vector, and the sequences of resulted plasmids were confirmed by DNA-sequencing analysis. Finally, expression plasmid using for transfection was prepared using a Plasmid Mini Kit (Qiagen), according to the manufacturer’s protocol.

### Cell culture

HEK293 cells were harvested in Dulbecco’s modified Eagle’s medium (DMEM; Sigma) supplemented with 10% (w/v) foetal bovine serum (Biosera) at 37 °C with 5% (v/v) CO_2_. Tet-ADAR2 cells, in which hADAR2 and AcGFP were simultaneously expressed from a bi-directional promoter under the control of the Tet-on system, were previously established in our laboratory. Tet-ADAR2 cells were cultured as monolayers in DMEM supplemented with 10% Tet system-approved foetal bovine serum (Clontech), 1 μg/mL puromycin (Sigma), and 100 μg/mL G418 (Sigma) at 37 °C in 5% (v/v) CO_2_. To induce ADAR2 and AcGFP expression, tet-ADAR2 cells were cultured in the above medium supplemented with 5 μg/mL of Dox.

### Analysis of intracellular editing-induction efficiency of AD-gRNAs

Tet-ADAR2 cells (1.6 × 10^5^) were added to 35-mm dishes in medium containing 5 μg/mL Dox. After culturing for 24 h, they were transfected with 2 μg of the AD-gRNA expression plasmid, using the X-tremeGENE HP DNA Transfection Reagent (Roche). At 72 h post-transfection, the editing efficiency at A200 in AcGFP RNA, which was simultaneously expressed with hADAR2 in Tet-ADAR2 cells, was analysed as follows. Total RNA was extracted from the transfected cells using Sepasol RNA I Super G (Nacalai Tesque), according to the manufacturer’s protocol. Then, RNA samples (30 μg) were treated with 10 U DNase I (Takara Bio) for 1 h at 37 °C, followed by phenol/chloroform extraction and ethanol precipitation. Purified RNA (0.5 μg) RNA was reverse transcribed using the adapter-linked oligo(dT) 17 primer and the Transcriptor High Fidelity cDNA Synthesis Kit (Roche), according to the manufacturer’s protocol. Using the obtained total cDNA as a template, AcGFP cDNA was amplified by PCR with AcGFP-specific primers (AcGFP_F and AcGFP_R). The efficiency of A-to-I RNA editing at the A200 site was analysed by direct sequencing, followed by quantification of the relative heights of the A and G peaks, after which each editing ratio was calculated on the basis of the peak height of G divided by that of A + G.

### Intracellular codon-repair experiments with AD-gRNAs

In the *in vitro* codon-repair experiments, the mutant GFP mRNA (AcGFP-W58X), in which G173 was changed to A173 to mutate codon W58 into a stop codon, was designed to determine whether AD-gRNA could regulate functional gene expression in cells. The AcGFP-W58X expression plasmid was constructed based on pcDNA3.1 expression vector (Invitrogen). In addition, hADAR2 expression plasmid was constructed by cloning PCR-amplified hADAR2 cDNA into pcDNA3.1. Seven hundred nanograms each of the hADAR2, AcGFP-W58X, and AD-gRNA expression vectors were co-transfected into sub-confluent HEK293 cells cultured in 35-mm glass bottom dishes (Falcon), using the X-tremeGENE HP DNA Transfection Reagent (Roche). The editing efficiency was analysed at 72 h post-transfection, and the recovery of intracellular GFP fluorescence was analysed by fluorescence microscopy.

## Additional Information

**How to cite this article**: Fukuda, M. *et al*. Construction of a guide-RNA for site-directed RNA mutagenesis utilising intracellular A-to-I RNA editing. *Sci. Rep.* **7**, 41478; doi: 10.1038/srep41478 (2017).

**Publisher's note:** Springer Nature remains neutral with regard to jurisdictional claims in published maps and institutional affiliations.

## Supplementary Material

Supplementary Data

Supplementary Table
