# Supplementary material for: Construction of a guide-RNA for site-directed RNA mutagenesis utilising intracellular A-to-I RNA editing
Source: Sci Rep. 2017 Feb 2;7:41478. doi: 10.1038/srep41478 (PMC5288656; doi:10.1038/srep41478)
Supplement: Supplementary Data [file srep41478-s1.pdf]

SUPPLEMENTARY INFORMATION

Construction of a guide-RNA for site-directed RNA mutagenesis utilising intracellular A-to-I RNA editing

Masatora Fukuda[1,]*, Hiromitsu Umeno[1], Kanako Nose[1], Azusa Nishitarumizu[1], Ryoma Noguchi[1] and Hiroyuki Nakagawa[2]

[1]Department of Chemistry and [2]Department of Earth System Science, Faculty of Science, Fukuoka University, Nanakuma 8-19-1, Jonan-ku, Fukuoka 814-0180, Japan

*E-mail: masatora@fukuoka-u.ac.jp

Supplementary Fig. S1

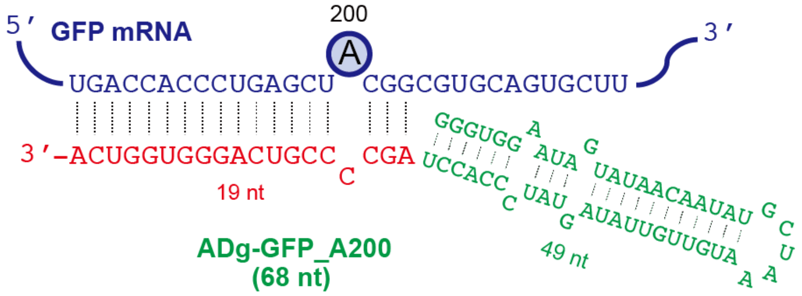

Supplementary Fig. S2

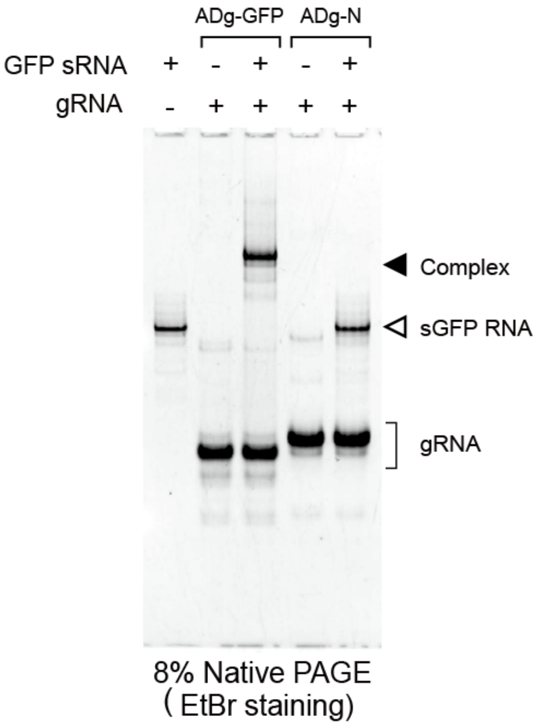

Supplementary Fig. S3

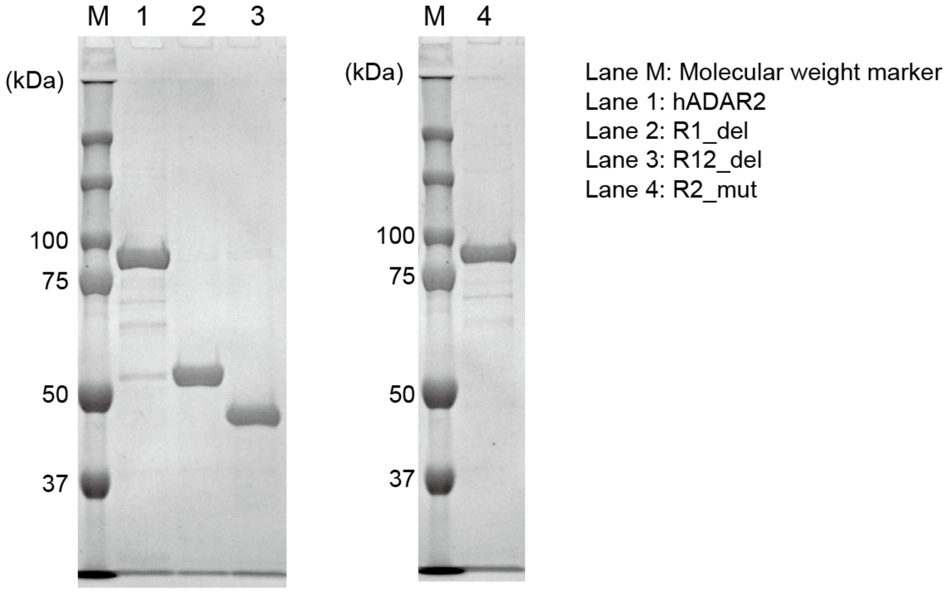

8% SDS-PAGE
(CBB staining)

Supplementary Fig. S4

(a) no guide

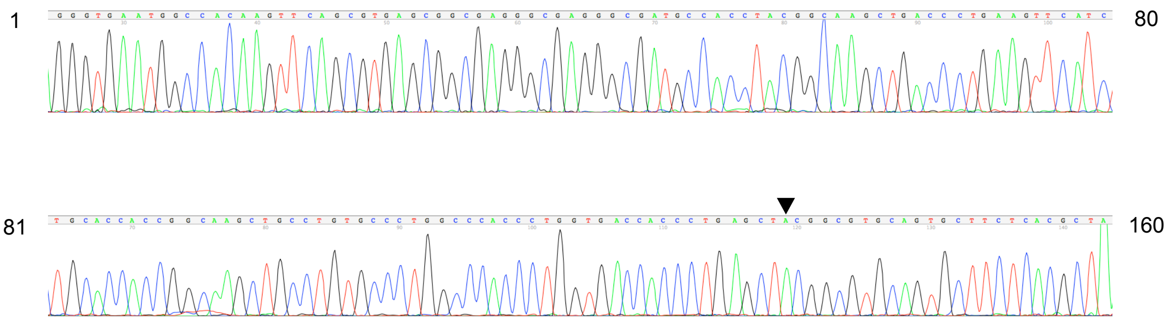

(b) 3′-AS-sGFP_A200

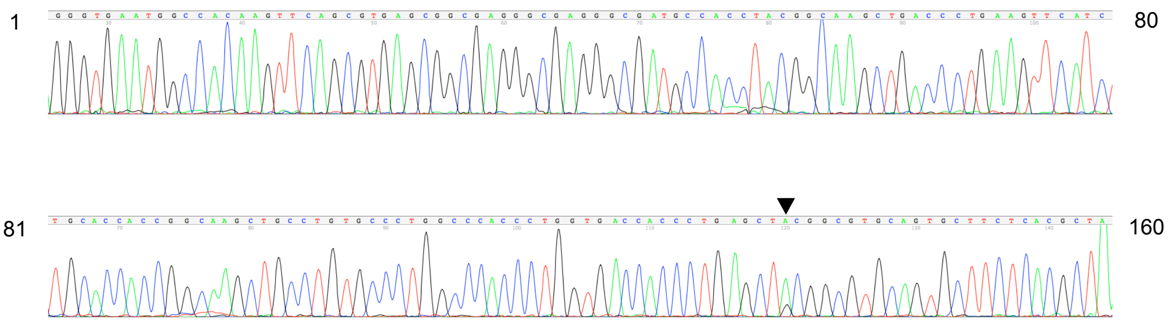

(c) ADg-sGFP_A200

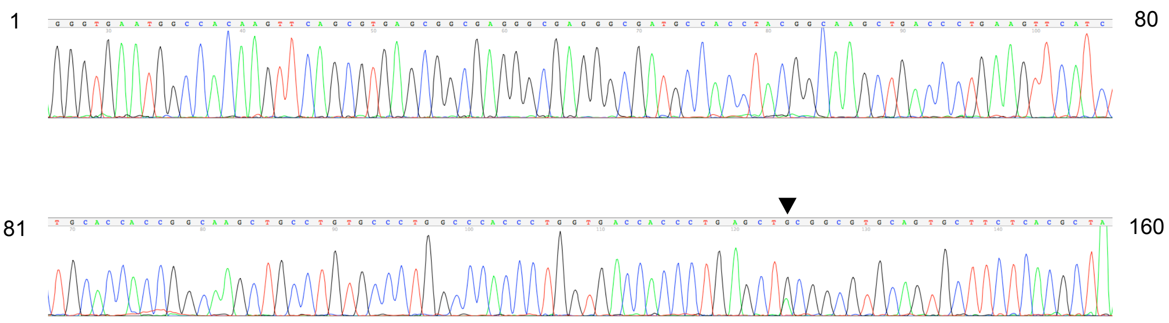

Supplementary Fig. S5

(a) no guide

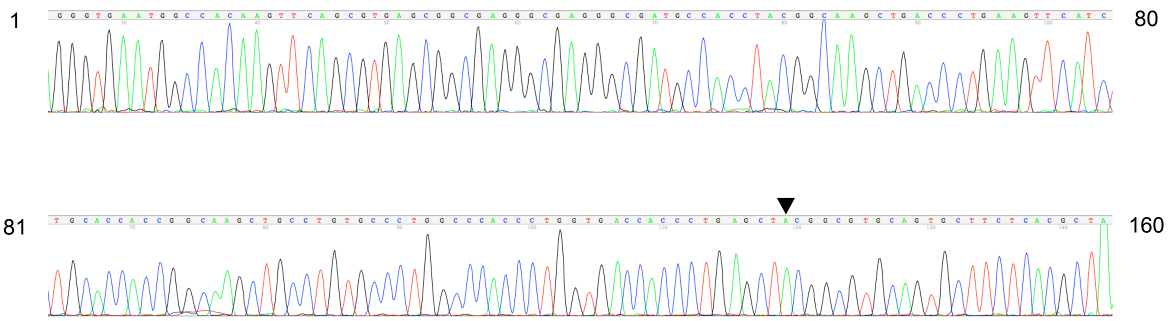

(b) 5′-AS-sGFP_A200

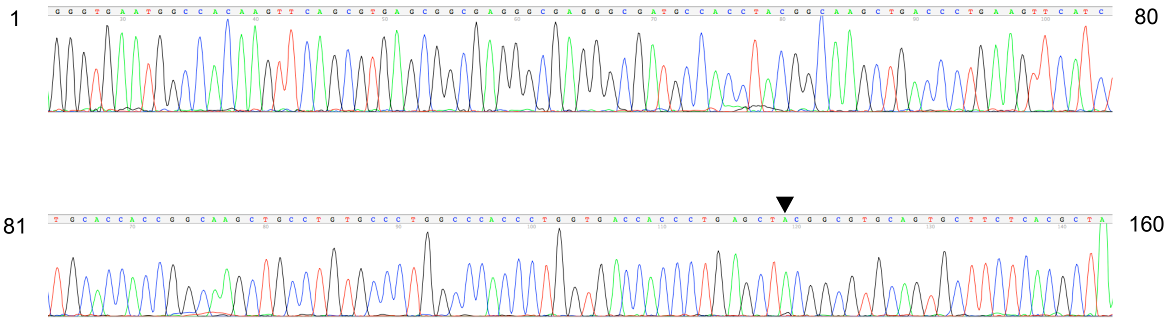

(c) ADg-rsGFP_A200

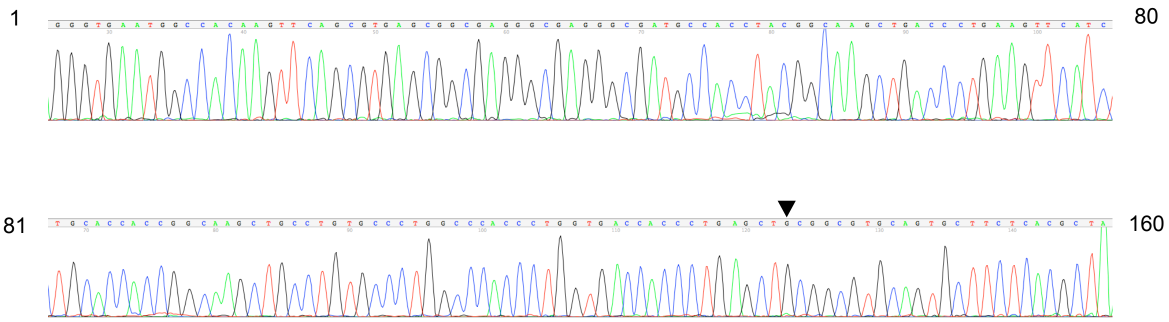

Supplementary Fig. S6

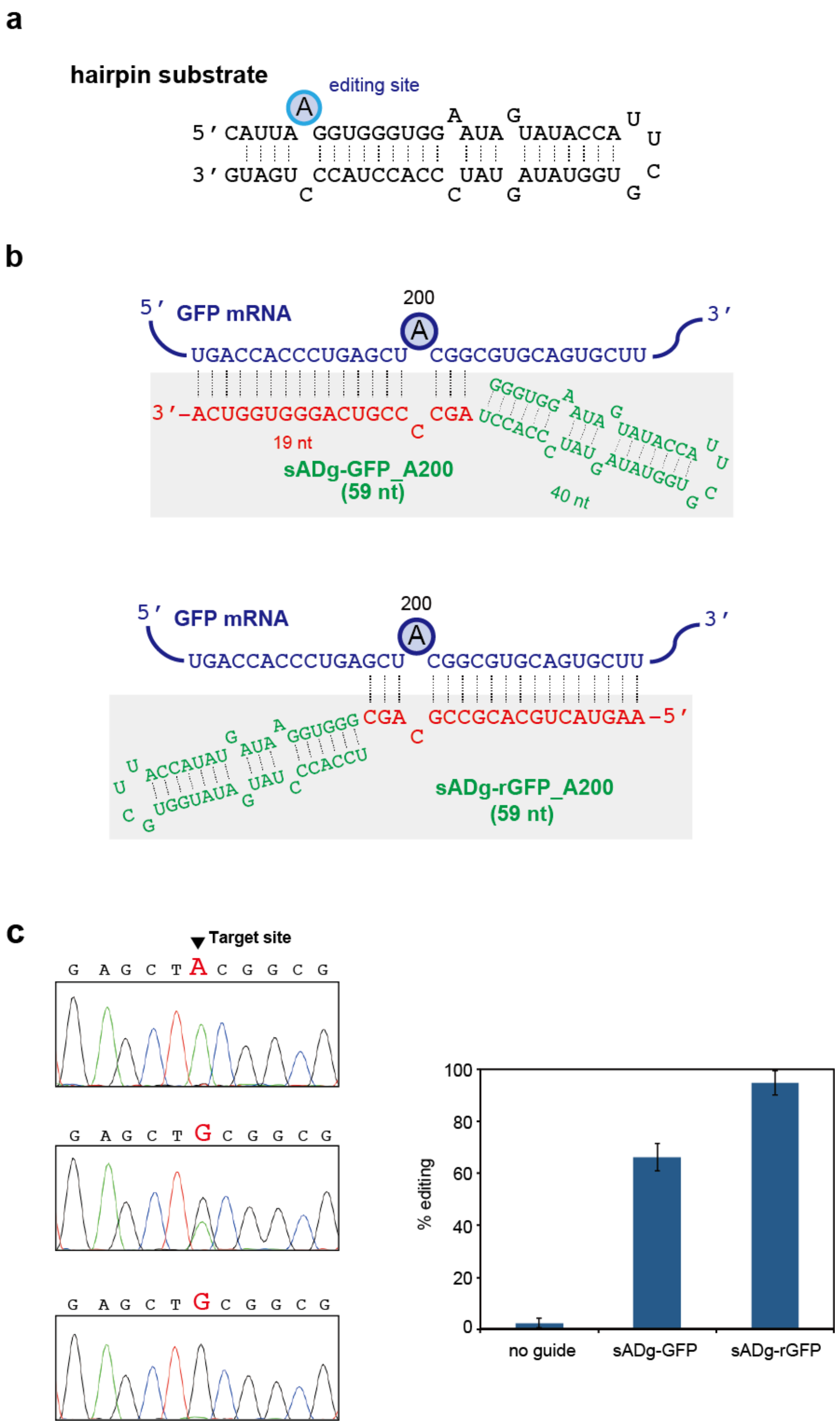

Supplementary Fig. S7

(a) sADg-sGFP_A200

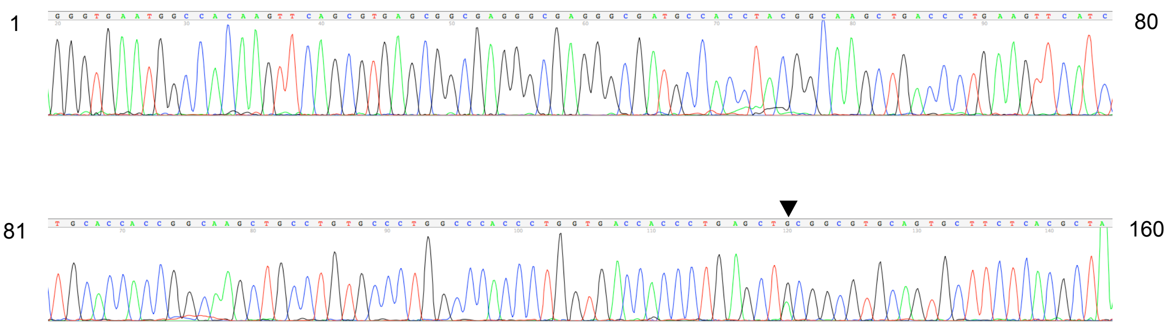

(b) sADg-rsGFP_A200

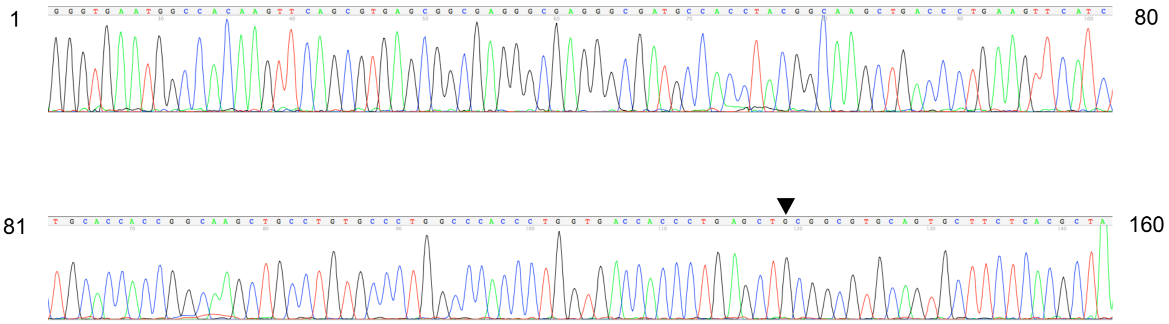

Supplementary Fig. S8

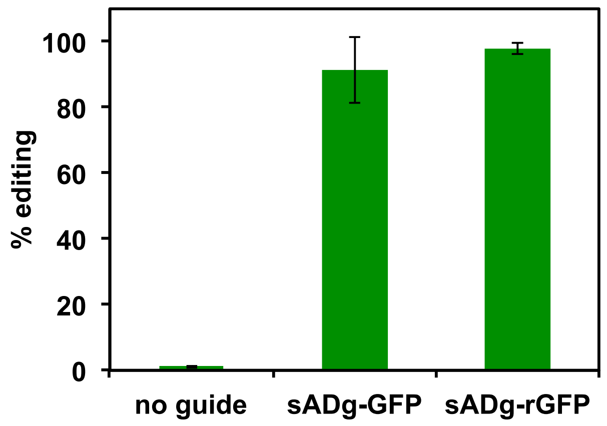

Supplementary Fig. S9

(a) no guide

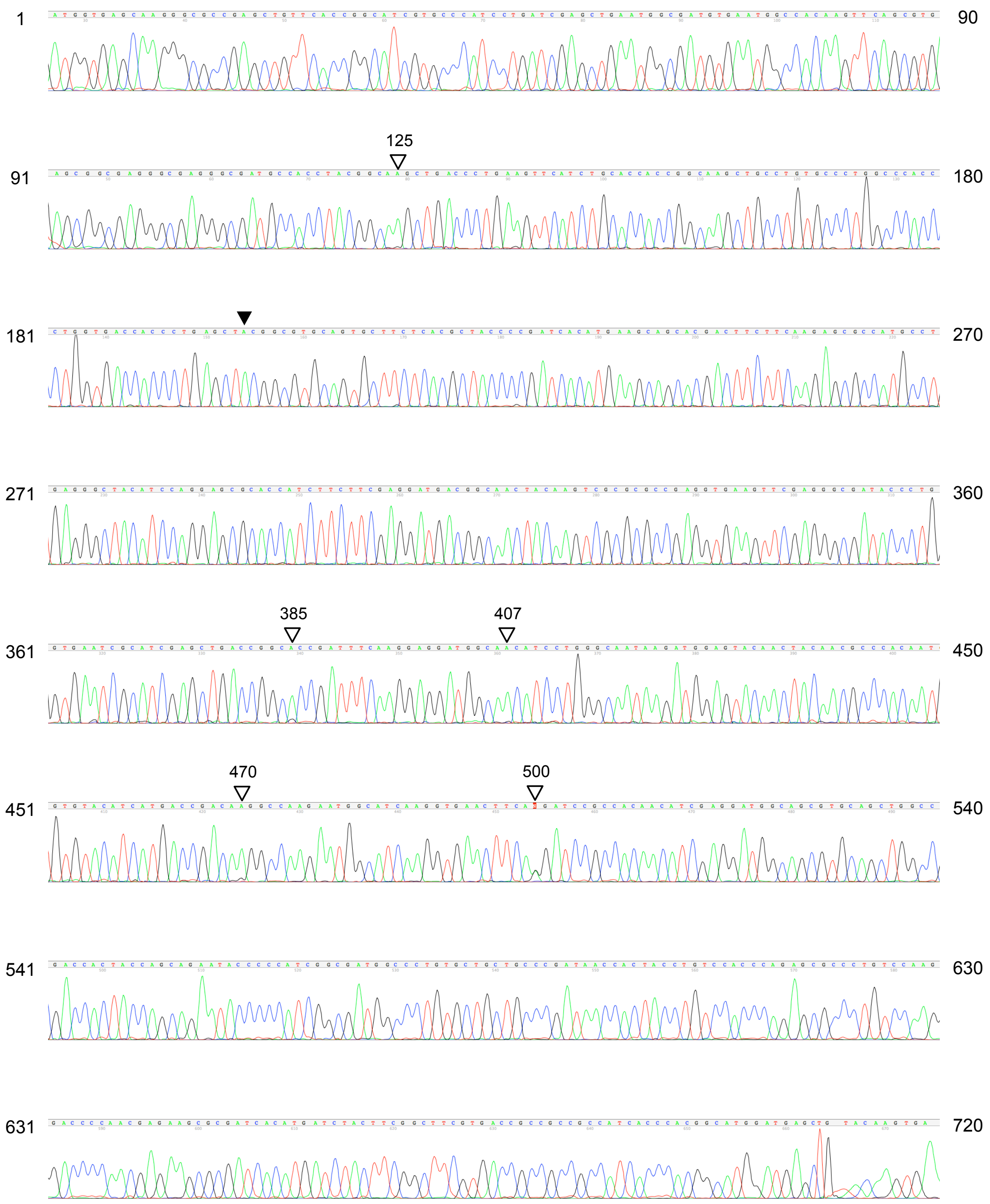

(b) sADg-GFP_A200

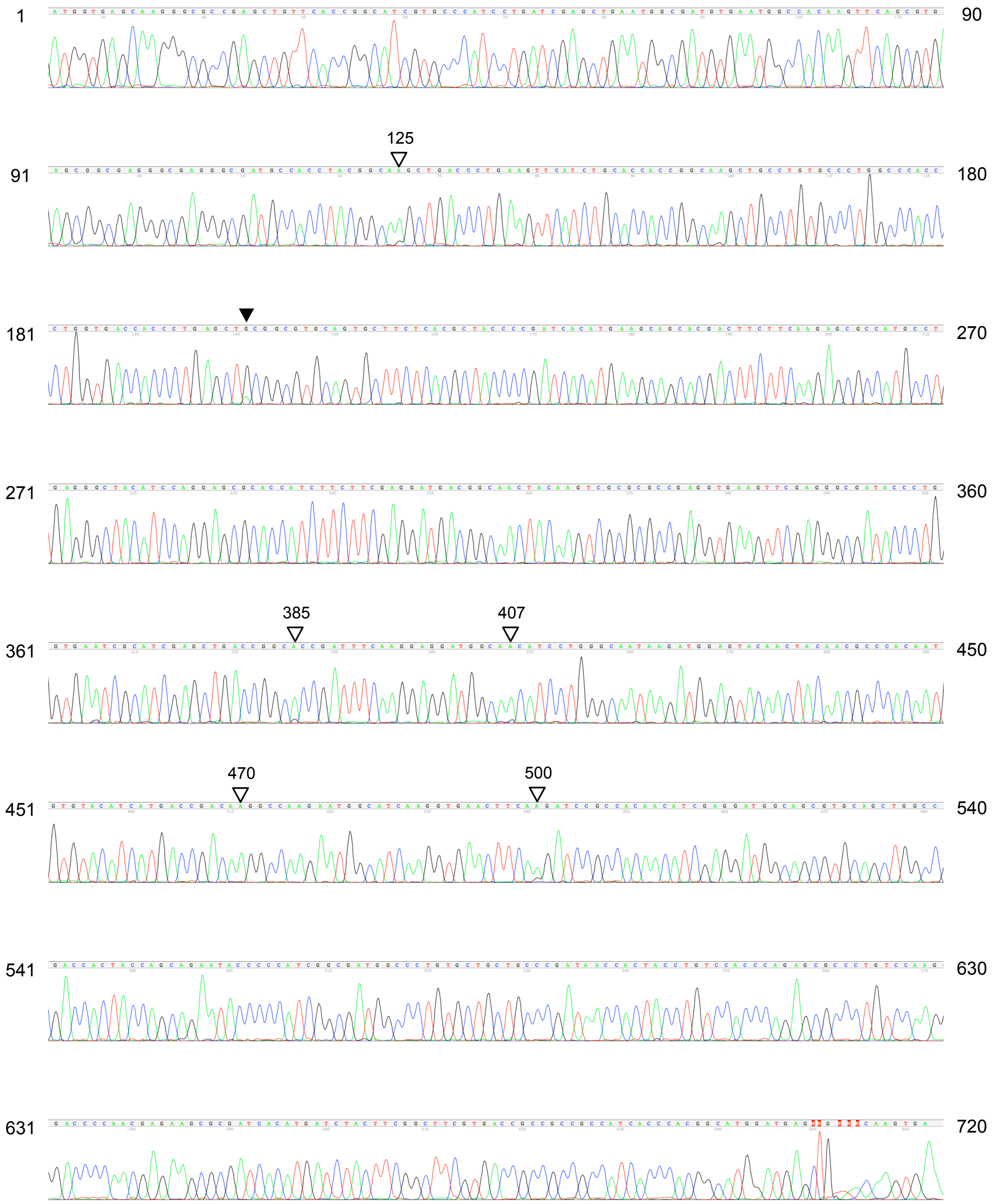

(c) sADg-rGFP_A200

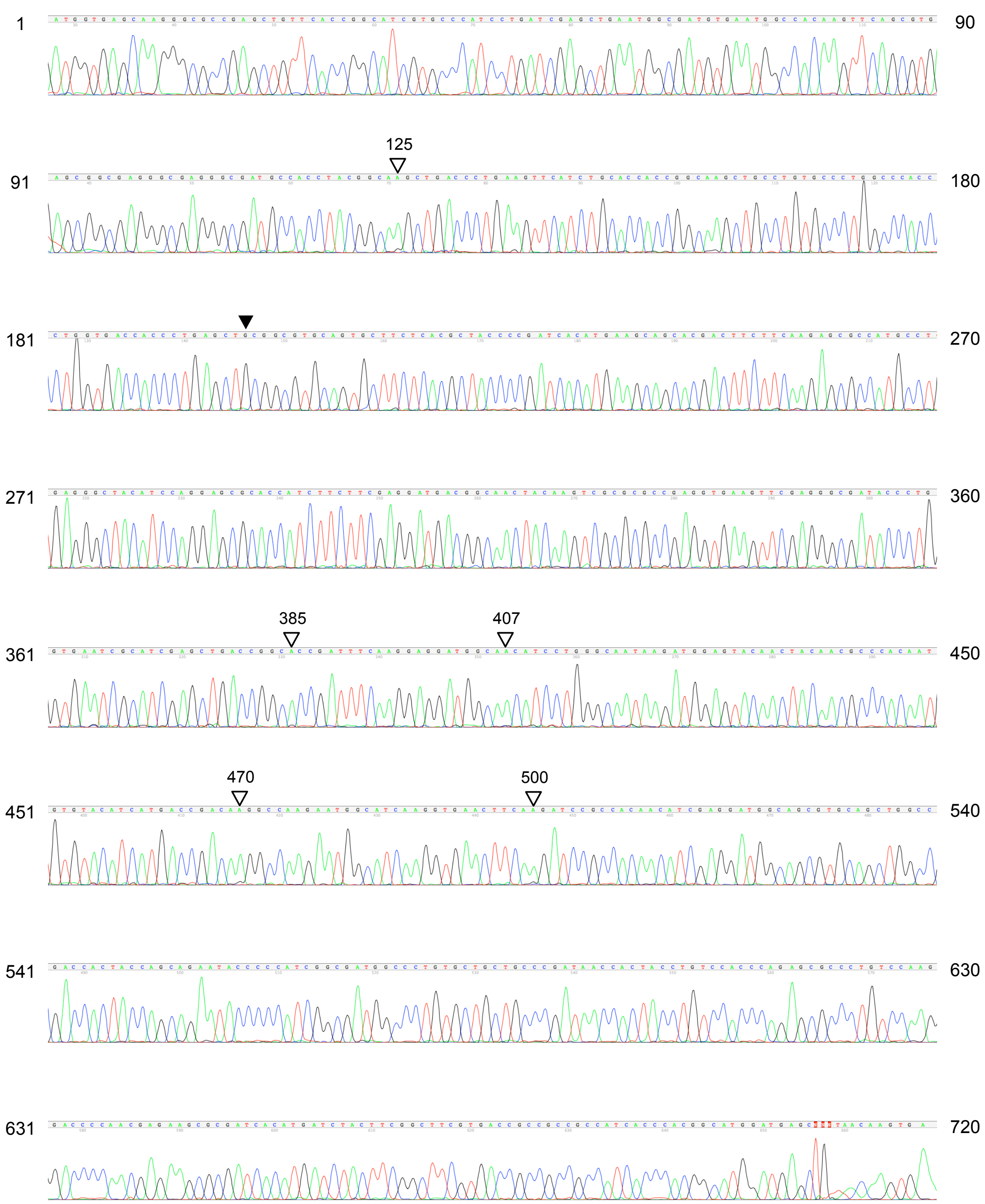

Supplementary Fig. S10

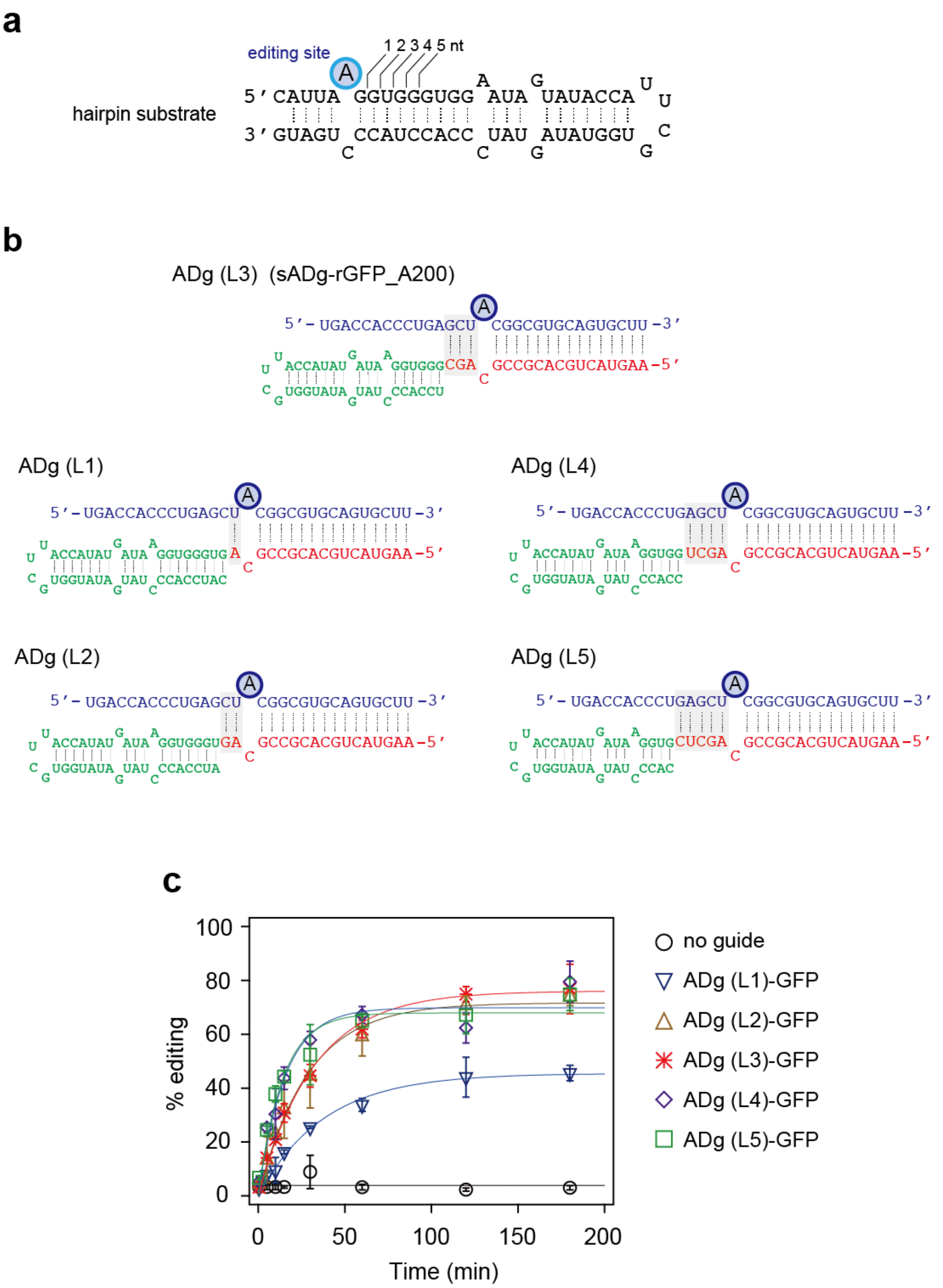

Supplementary Fig. S11

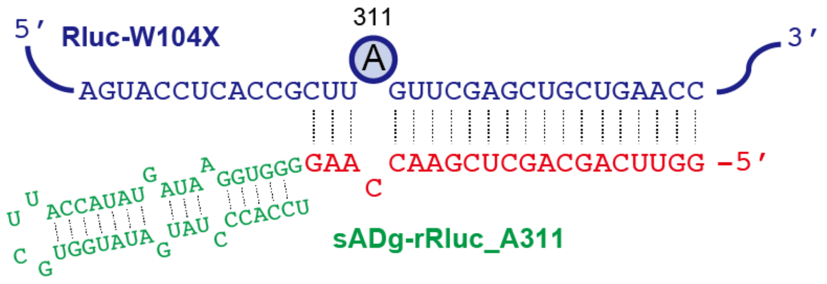

Supplementary Fig. S12

(a) Rluc WT ADAR (-) ADg-RNA (-)

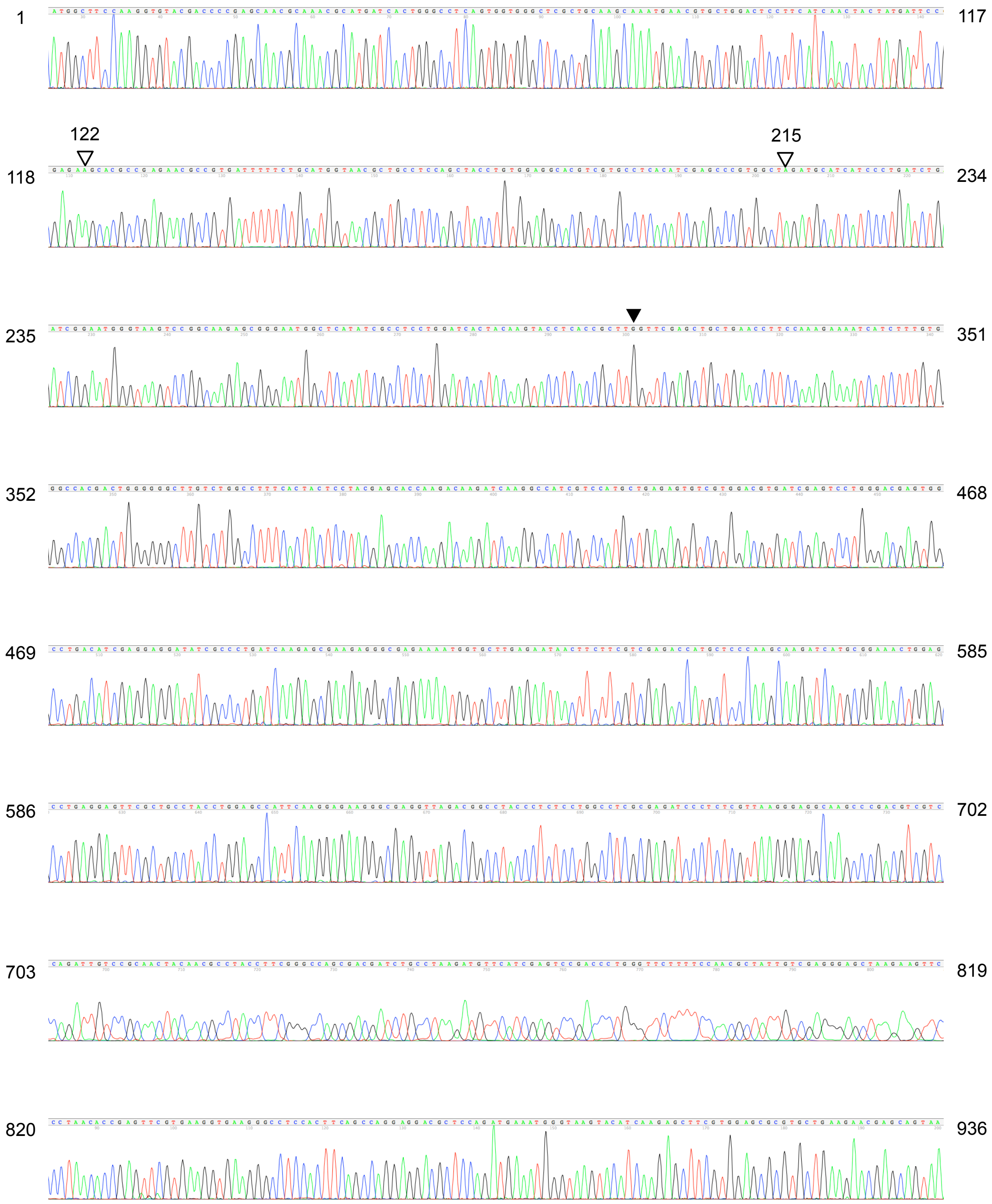

(b) Rluc WT ADAR (+) ADg-RNA (-)

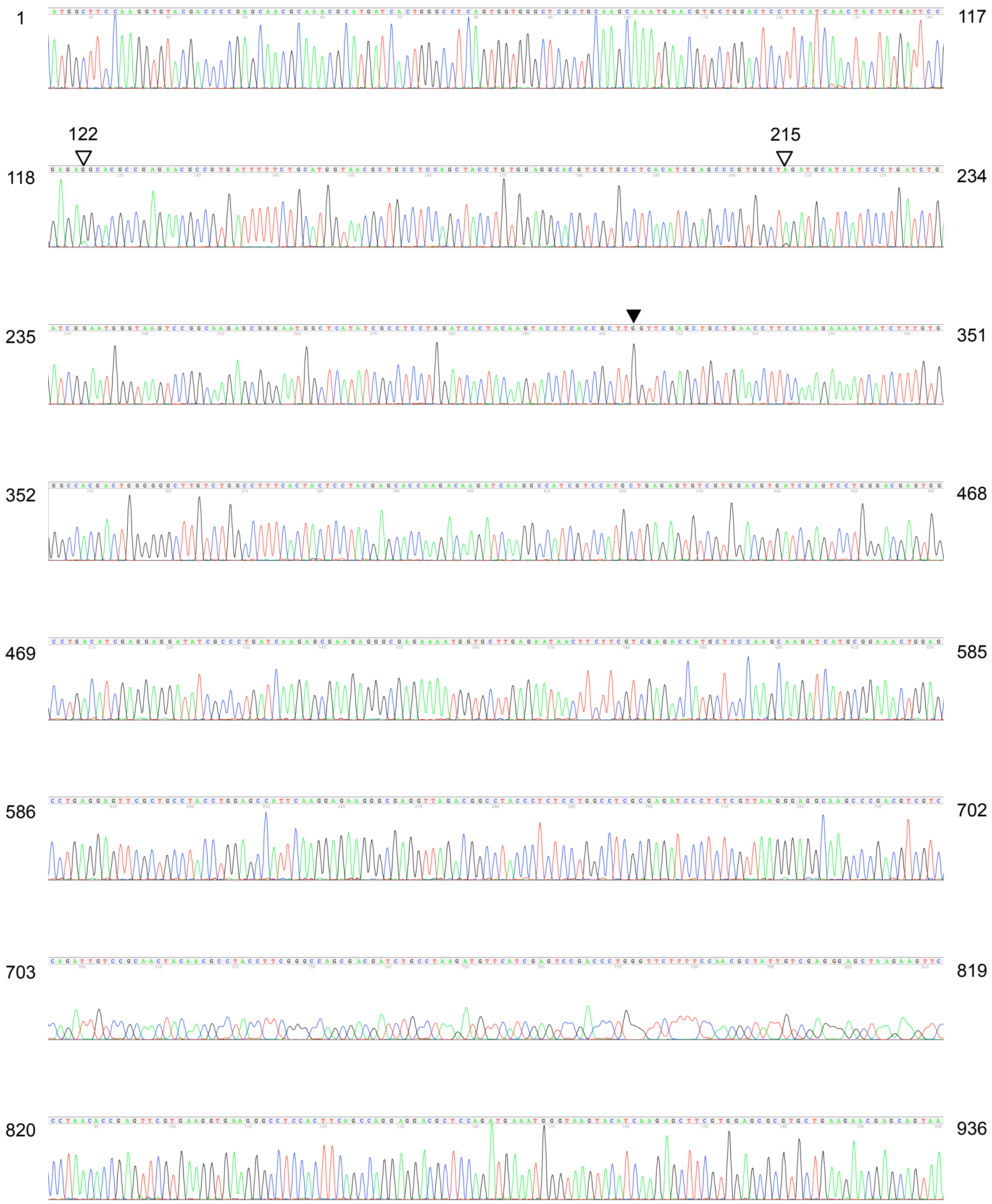

(c) Rluc W104X ADAR (+) ADg-RNA (-)

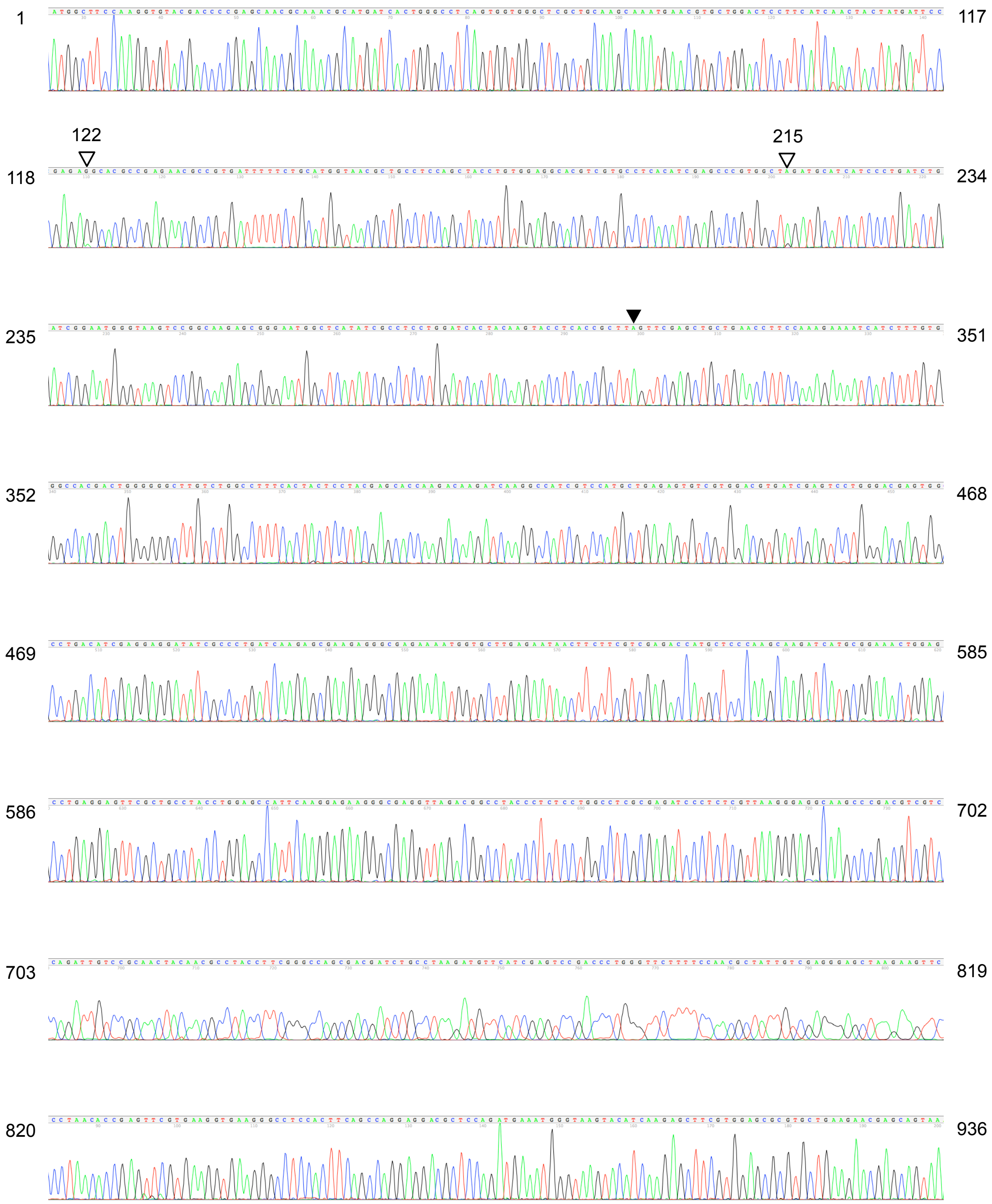

(d) Rluc W104X ADAR (+) ADg-RNA (+)

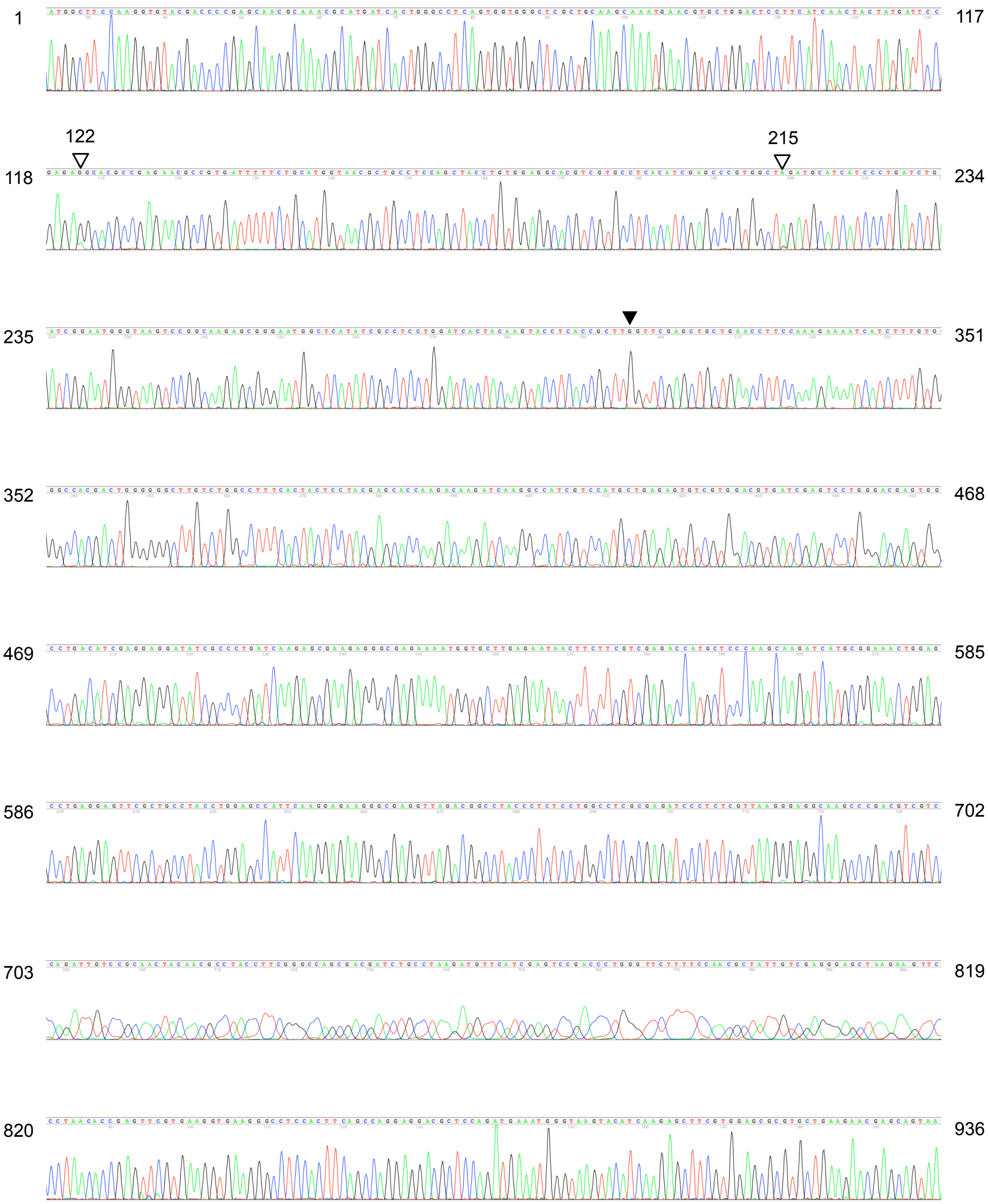

Supplementary Fig. S13

GAPDH

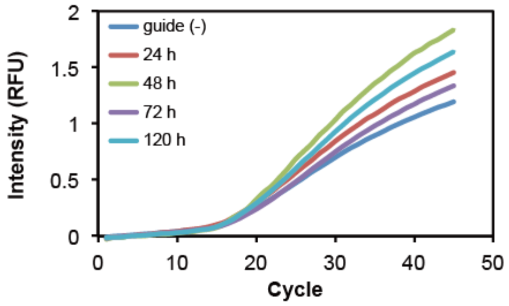

ADg-rGFP_A200

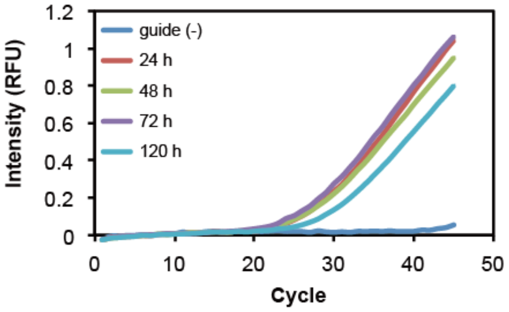

	Ct-value	
time (h)	GAPDH	AD-gRNA
24	16.197	25.575
48	16.249	27.658
72	17.376	26.578
120	16.478	30.463

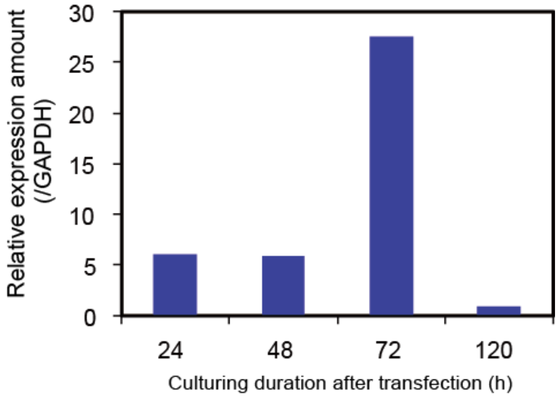

Supplementary Fig. S14

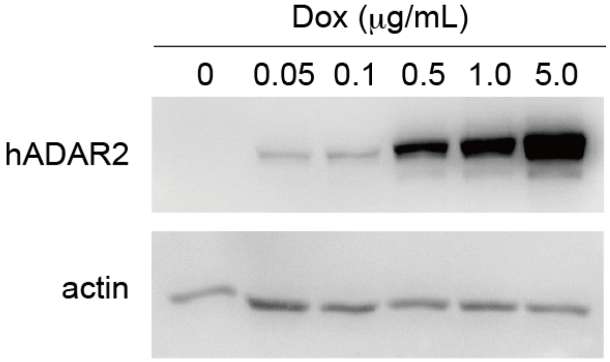

Supplementary Fig. S15

(a) FLNA Q/R site

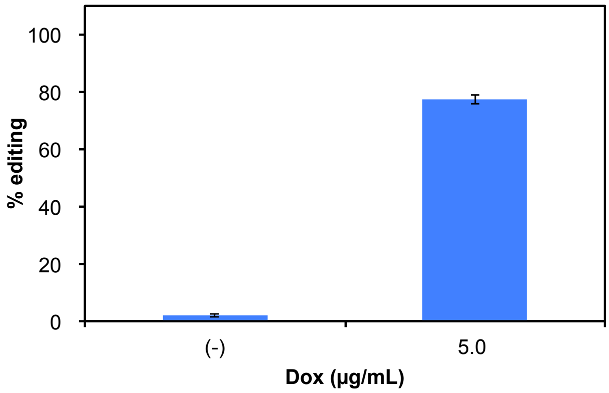

(b) BLCAP Y/C site

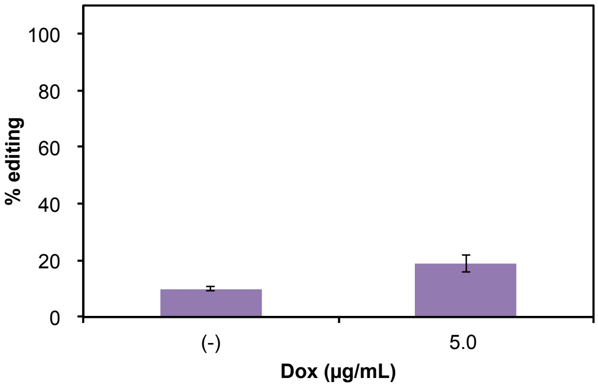

Supplementary Fig. S16

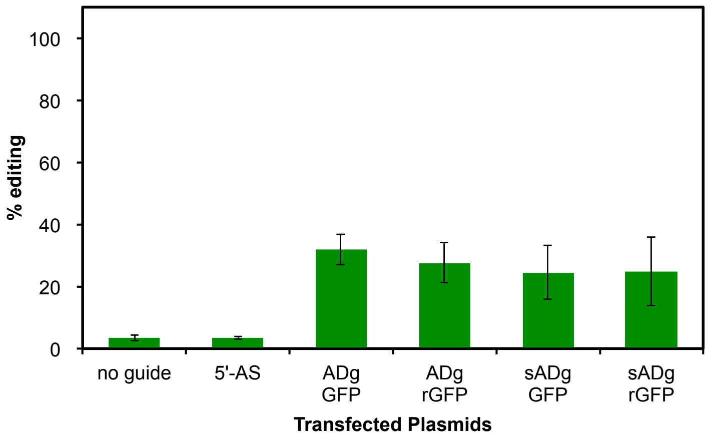

Supplementary Fig. S17

(a) Without Transfection

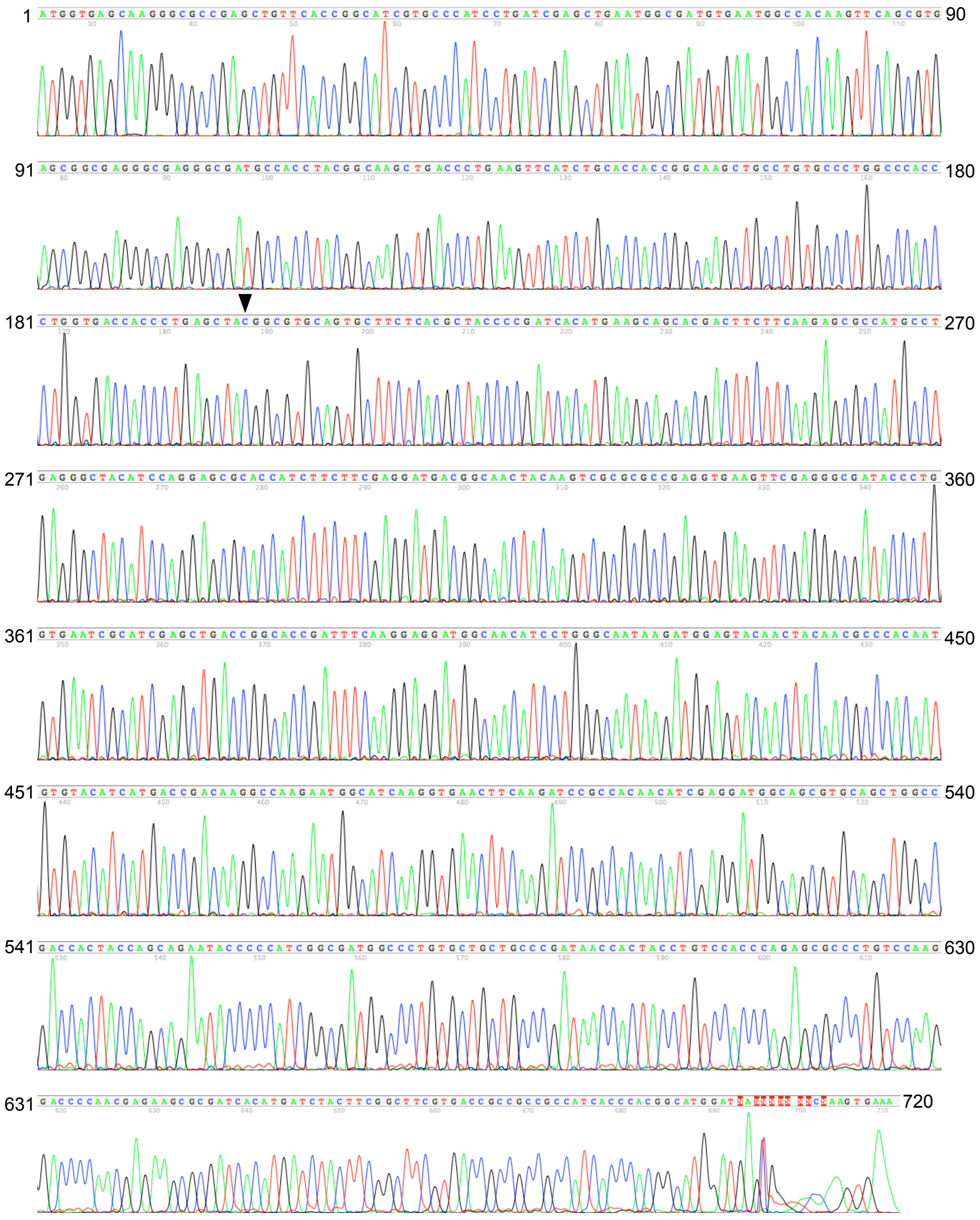

(b) 5′-AS

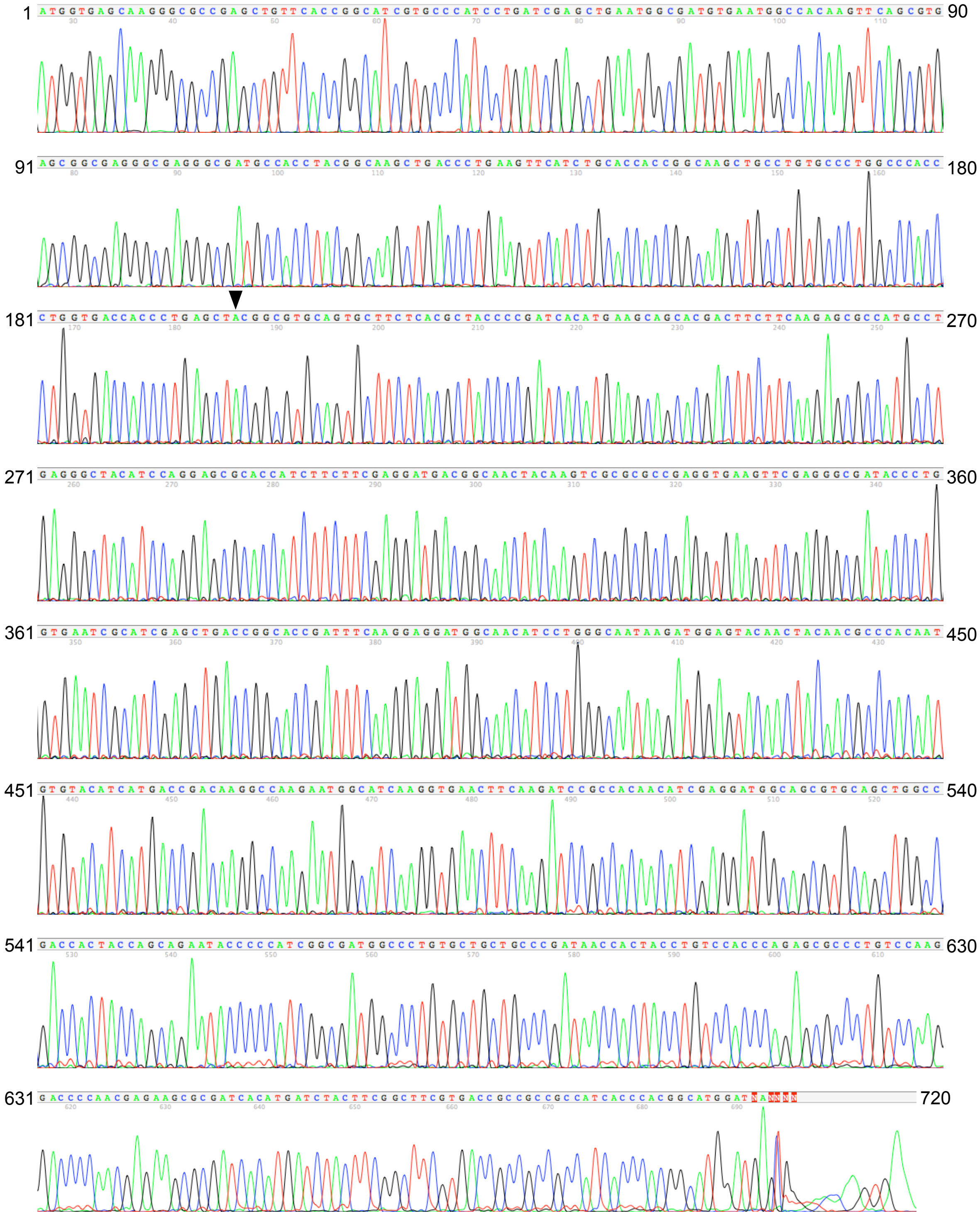

(c) ADg-GFP_A200

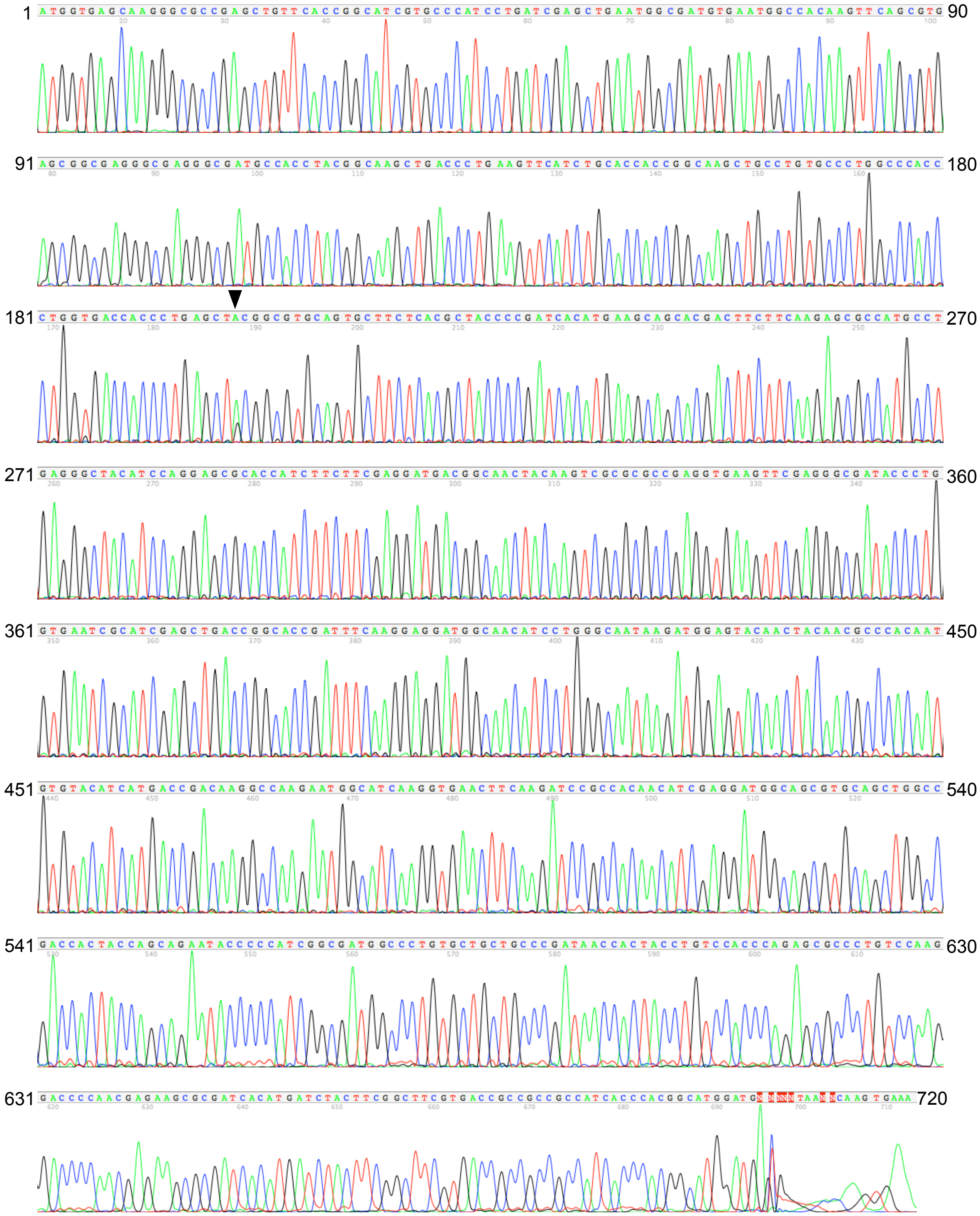

(d) ADg-rGFP_A200

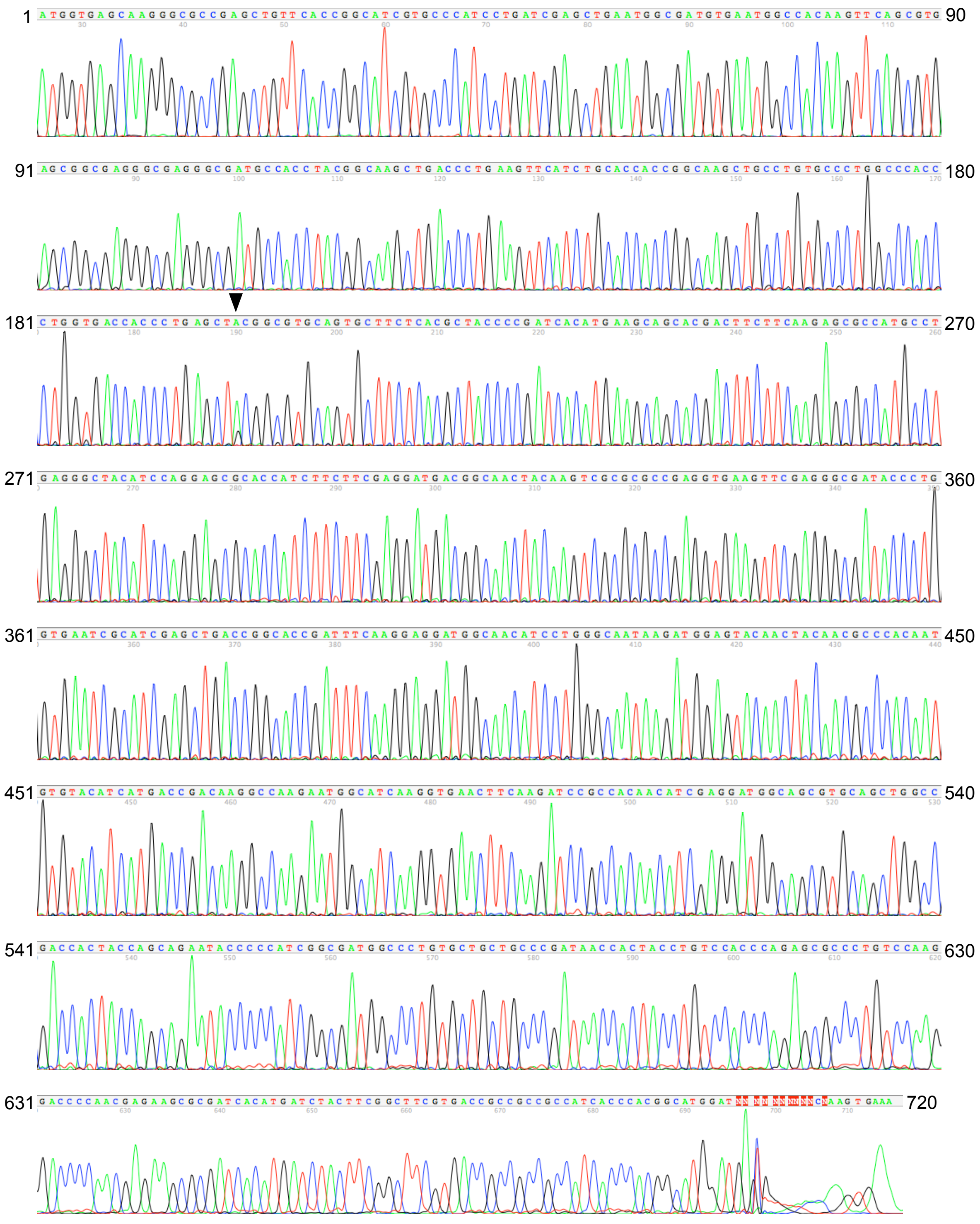

(e) sADg-GFP_A200

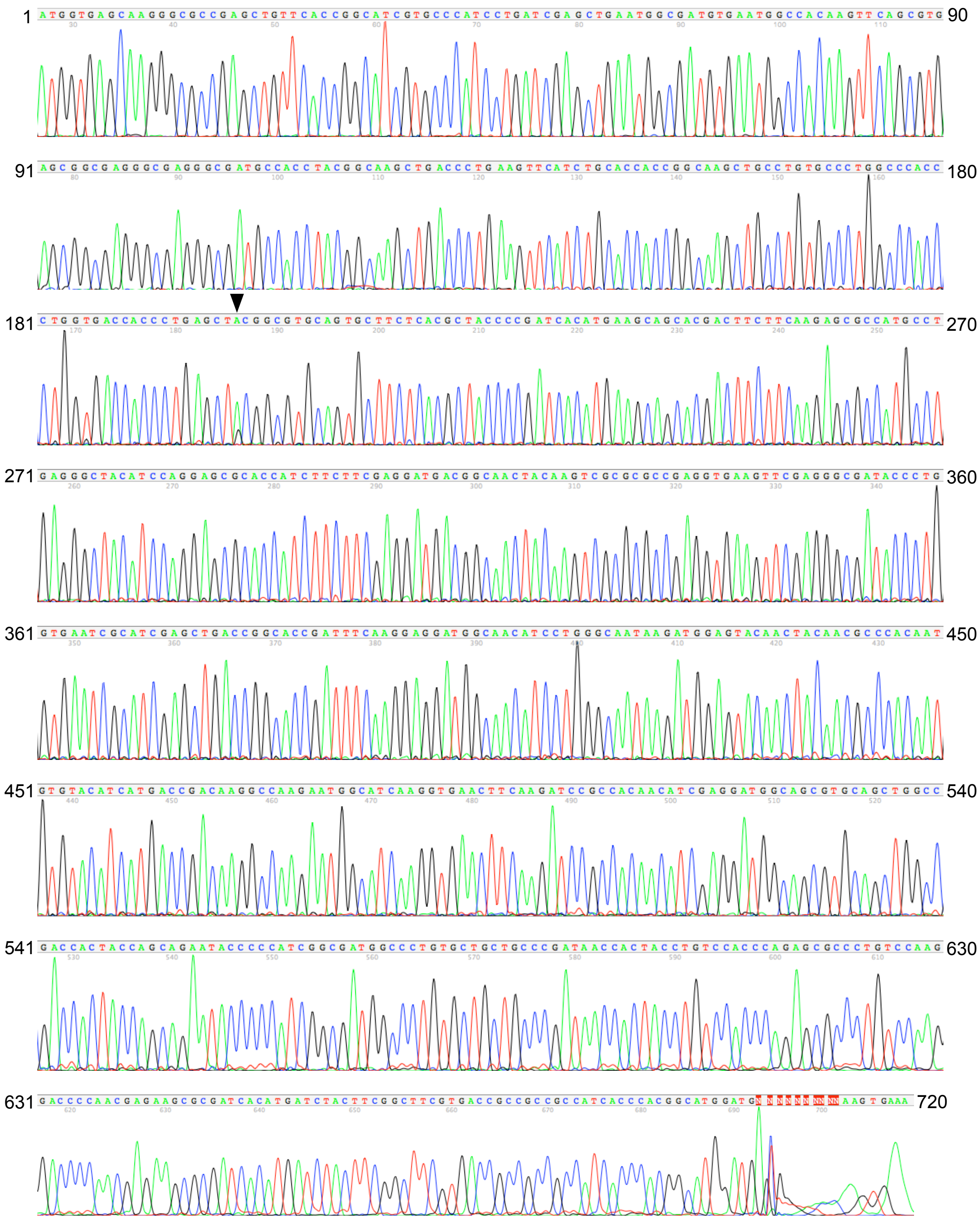

(f) sADg-rGFP_A200

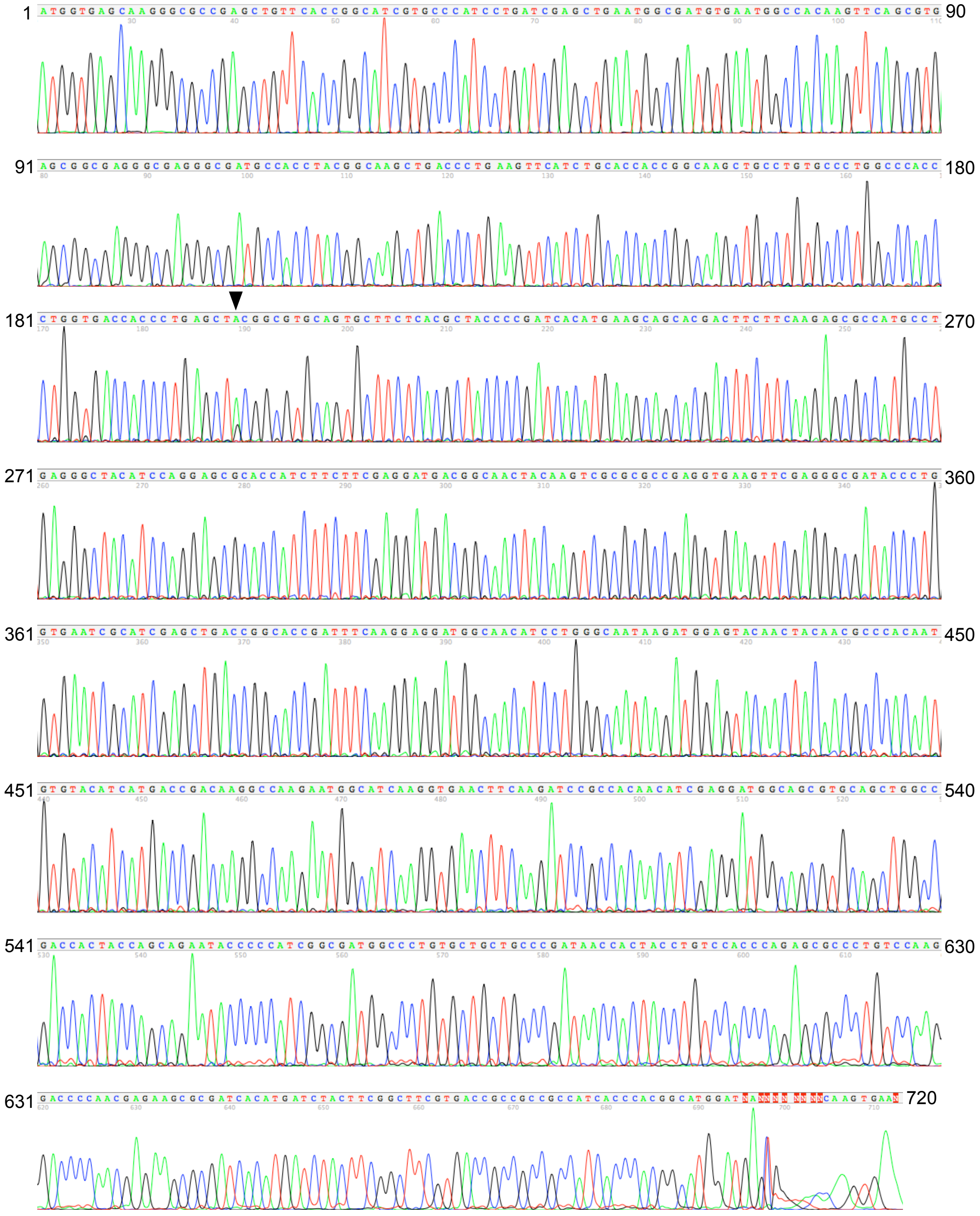

Supplementary Fig. S18

(a) FLNA Q/R site

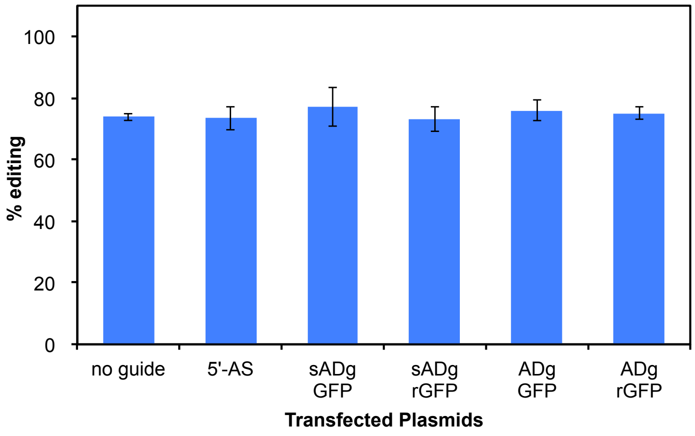

(b) BLCAP Y/C site

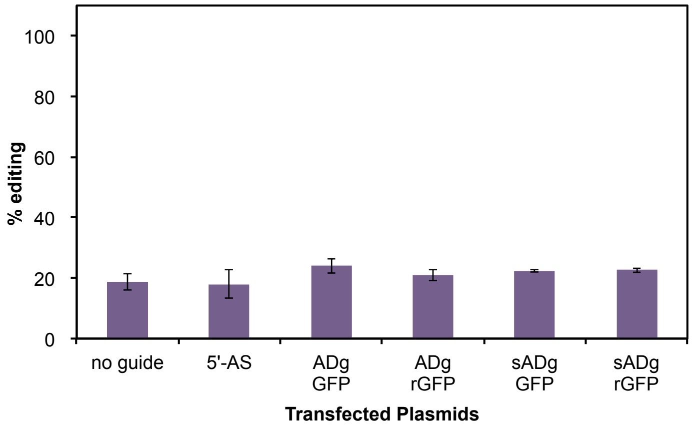

Supplementary Fig. S19

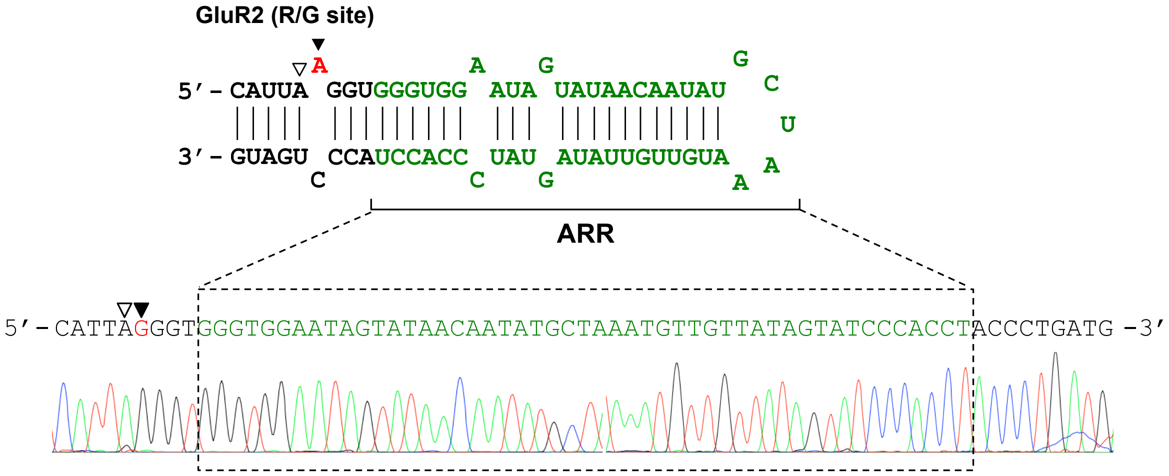

Supplementary Fig. S20

(a) GFP_A173

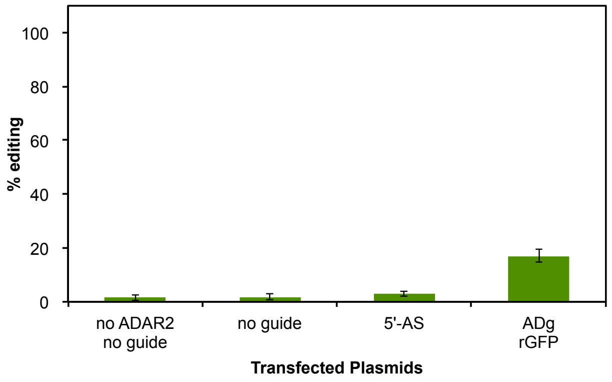

(b) FLNA Q/R site

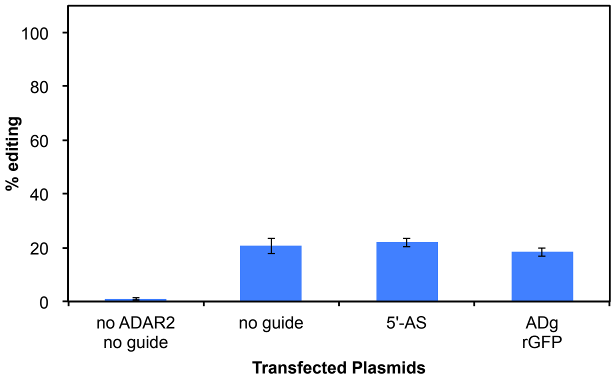

(c) BLCAP Y/C site

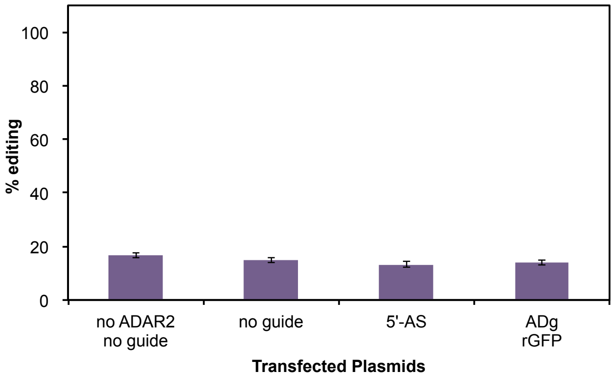

Supplementary Figure S21

(a) non-transfected HEK293 cells

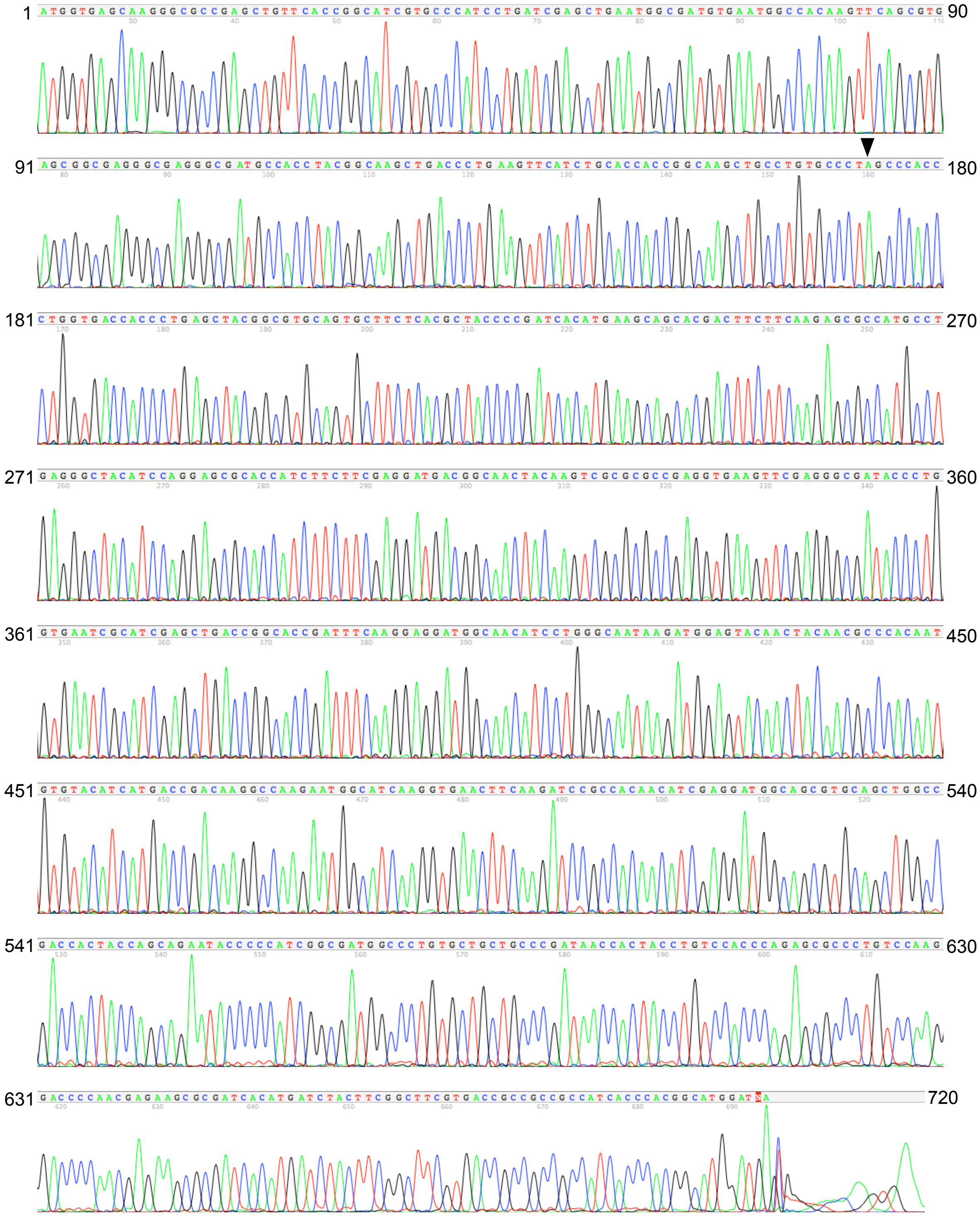

(b) Without co-transfection of the expression plasmid

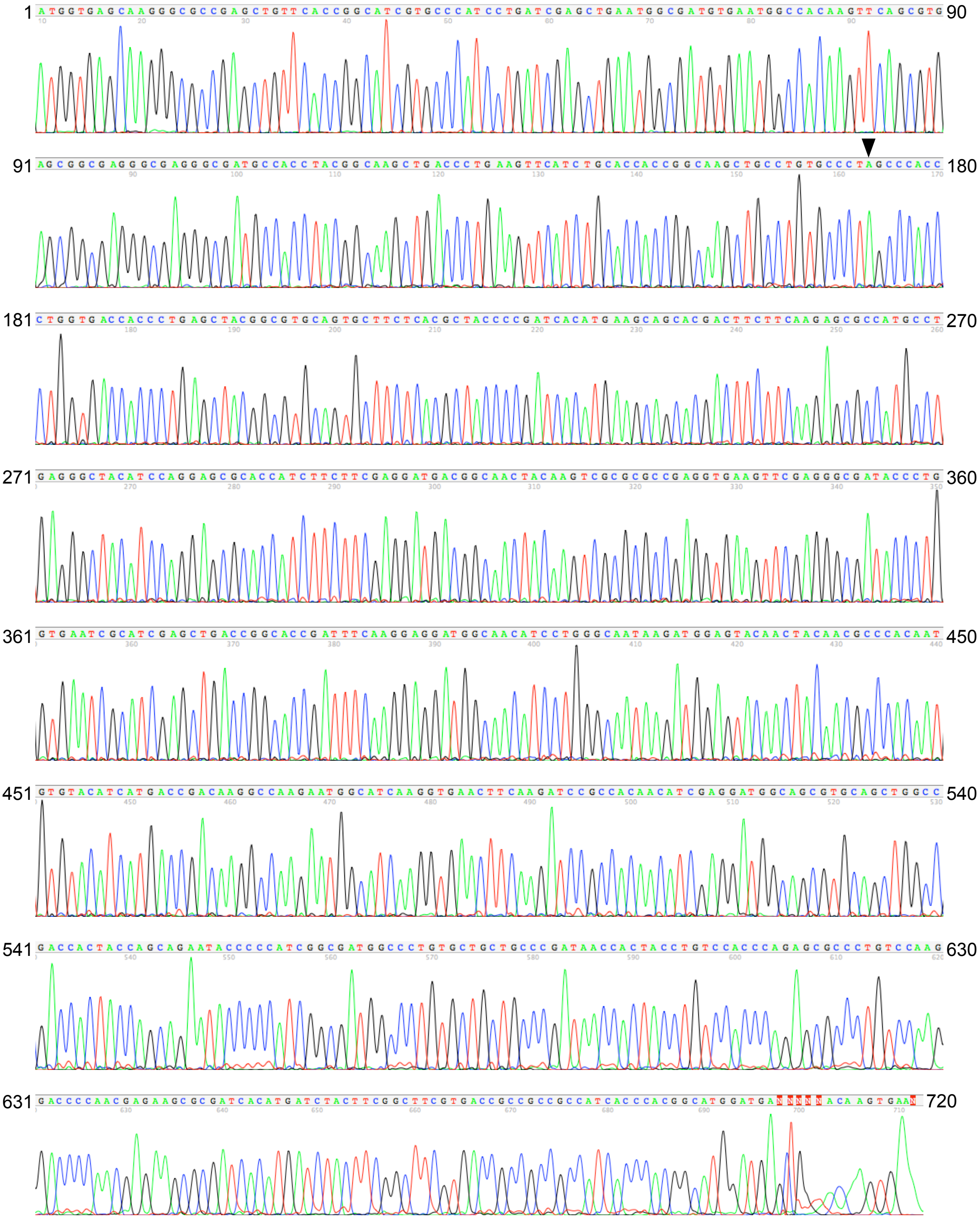

(c) co-transfection with p-5′-AS

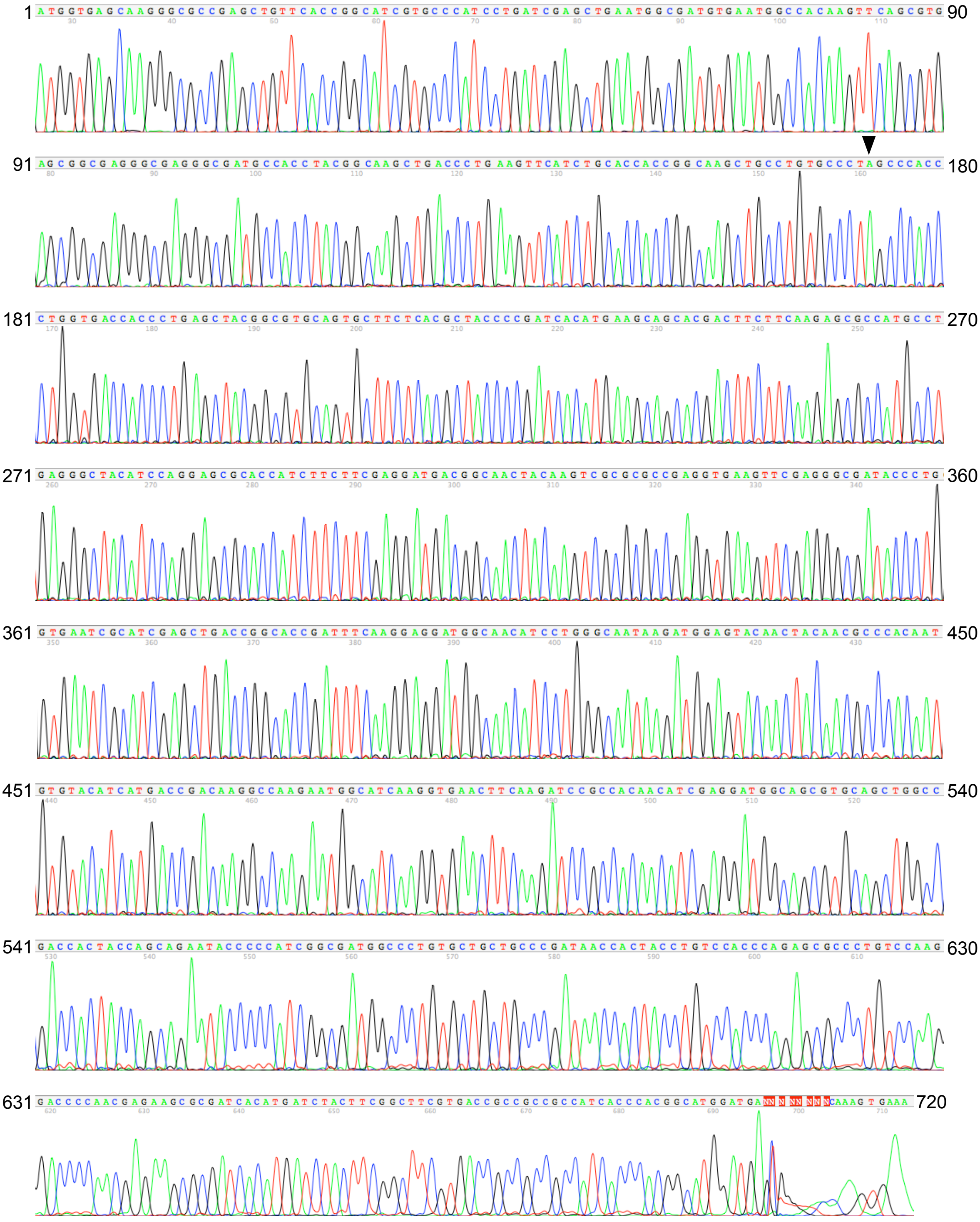

(d) co-transfection with p-ADg-rGFP_A173

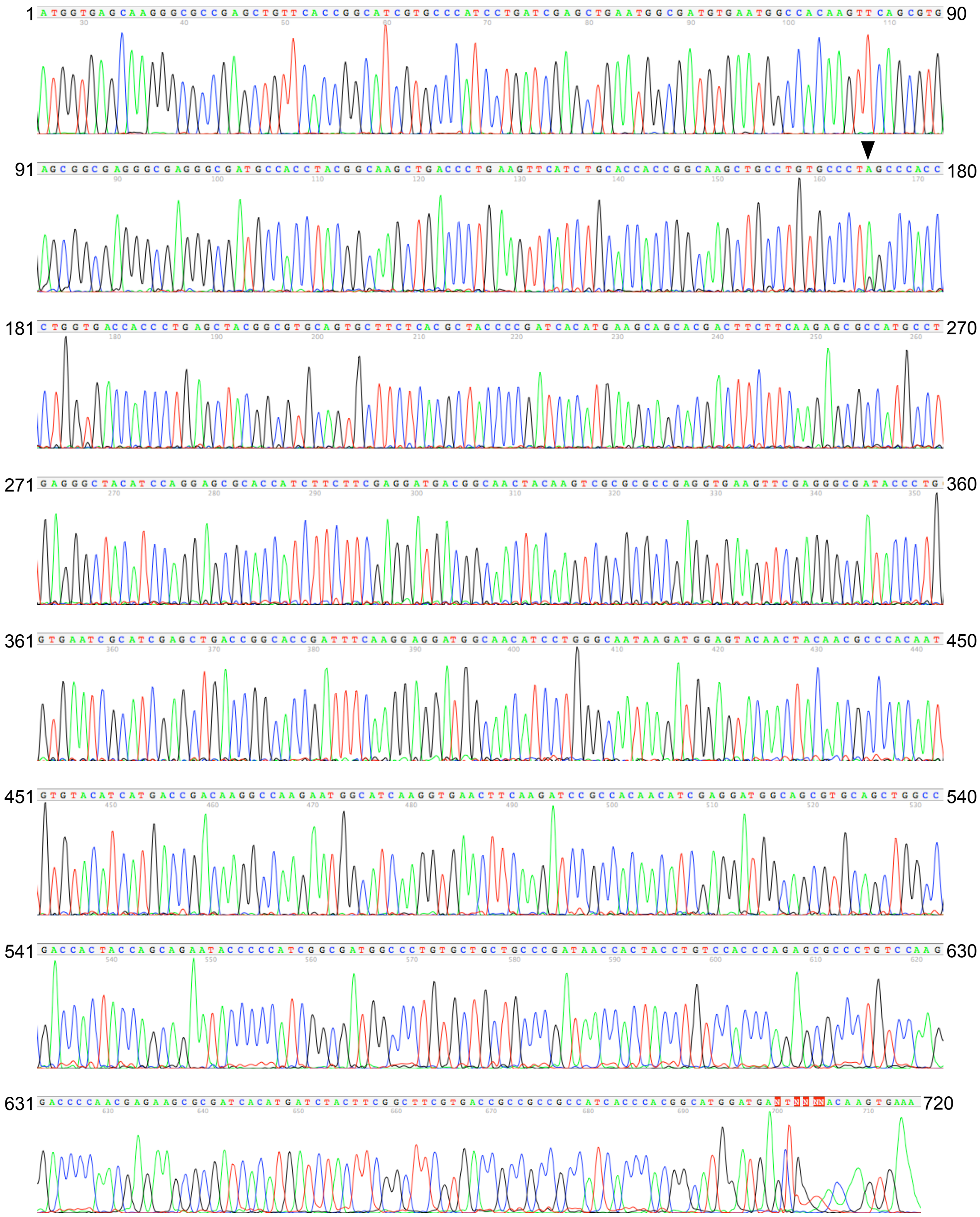

Supplementary Figure 1

Sequence design of ADg-GFP_A200. Nucleotides sequences of ADg-GFP_A200 and partial sequence of sGFP RNA are shown as a complex with predicted secondary structures. sGFP RNA is represented in blue, and the target-editing site (A200) is depicted with a circled 'A'. The antisense region (ASR) and ADAR-recruiting region (ARR) are shown in red and green characters, respectively.

Supplementary Figure 2

Gel-mobility shift assay for complex formation with AD-gRNA and target RNA. Each sample was prepared by an annealing reaction, using the oligonucleotide combinations indicated for each lane. ADg-GFP_A200 is represented by ADg-GFP. 'ADg-N' refers to a non-specific AD-gRNA that was constructed with an identical ARR and the 16 nt non-specific ASR (5'-GGCCGACAAUACUUUC-3'). The bands corresponding to free sGFP RNA and AD-gRNAs are indicated by the white arrowhead and bracket, respectively. Shifted band generated by the annealing reaction are indicated by the black arrowhead.

Supplementary Figure 3

Sodium dodecyl sulphate-polyacrylamide gel electrophoresis (SDS-PAGE) analysis of purified ADAR2 and its mutants used in this study.

Supplementary Figure 4

Entire sequencing chromatograms of sGFP RNA after the editing reaction with ADg-GFP_A200. The sequencing chromatograms shown were obtained by performing editing reactions with (**a**) ADAR2, (**b**) ADR2 + 3′-AS, and (**c**) ADAR2 + ADg-GFP_A200.

Supplementary Figure 5

Entire sequencing chromatograms of sGFP RNA after the editing reaction with ADg-rGFP_A200. The sequencing chromatograms shown were obtained by the editing reaction with (**a**) ADAR2, (**b**) ADAR2 + 5′-AS, and (**c**) ADAR2 + ADg-GFP_A200.

Supplementary Figure 6

Construction of short AD-gRNA based on the hairpin substrate. (**a**) Sequence and predicted secondary structure of the hADAR2 substrate hairpin structure. (**b**) Nucleotide sequence of sADg-GFP_A200 (upper) and sADg-rGFP_A200 (lower) representing the predicted secondary structures of the complex formed with sGFP RNA. (**c**) In vitro editing-induction activity of sADg-GFP_A200 and sADg-rGFP_A200. Sequencing chromatograms obtained by the editing reaction, without gRNA (upper panel), sADg-GFP_A200 (middle), or sADg-rGFP_A200 (lower) are shown. Editing ratios calculated from A and G peak heights are shown in the bar graph. The results are presented as averages and standard deviations from 3 independent experiments.

Supplementary Figure 7

Entire sequencing chromatograms of sGFP RNA after the editing reaction with sAD-gRNA. The sequencing chromatograms shown for sGFP cDNA were obtained after performing editing reactions with (**a**) ADAR2 + sADg-GFP_A200 and (**b**) ADAR2 + sADg-rGFP_A200.

Supplementary Figure 8

Editing-induction efficiency of sAD-gRNA at A200 using the full-length GFP mRNA template

Supplementary Figure 9

Entire sequencing chromatograms of full-length GFP mRNA after the editing reaction with sAD-gRNA. The sequencing chromatograms shown were obtained after performing editing reactions (**a**) without ADAR2, or with (**b**) ADAR2, (**c**) ADAR2 + sADg-GFP_A200, or (**d**) ADAR2 + sADg-rGFP_A200. Target and off-target editing sites are indicted with black and white arrowheads, respectively.

Supplementary Figure 10

Editing efficiency of sAD-gRNAs generated after dividing the template at various sites on the hairpin substrate. (**a**) Dividing line on the hairpin substrate. (**b**) Nucleotide sequence of 5′-AS sADg-RNAs, with various ARRs generated by dividing at the sites indicated in (**a**), represented as a complex with target-RNA (sGFP RNA). The ADg(L3) AD-gRNA was identical to sADg-rGFP_A200, which was used in the

experiment shown in Supplementary Fig. 6. (**c**) The editing-inducing activity of each sAD-gRNA. Changes in the editing efficiency at A200 at each reaction time are plotted in the graph. The data are presented as averages from 2 independent experiments.

Supplementary Figure 11

Sequence design of ADg-rRluc_A311. Nucleotide sequences of sADg-rRluc_A311 and the partial sequence of Rluc mRNA are shown as a complex with a predicted secondary structure. Rluc mRNA is represented in blue, and the target-editing site (A311) is depicted by the circled 'A'.

Supplementary Figure 12

Entire sequencing chromatograms of Rluc mRNA after the editing reaction with ADg-rRluc_A311. The represented sequencing chromatograms of Rluc cDNA were obtained by performing editing reactions (**a**) without ADAR2, or with (**b**) ADAR2 or (**c**) ADAR2 + ADg-rRluc_A311. Target and off-target editing sites are indicated by black and white arrowheads, respectively.

Supplementary Figure 13

RT and quantitative PCR (qPCR) analysis of ADg-GFP_A200 expressed in tet-ADAR2 cells. Amplification plots of GAPDH and ADg-rGFP_A200 at the indicated times post-transfection are shown graphically. The crossing threshold (Ct) values were calculated from each amplification plot by the second-derivative maximum method and are shown in the table. Differences in crossing thresholds (ΔCts) were calculated for each sample using the equation ΔCt = Ct (AD-gRNA) − Ct (GAPDH), and the relative expression level of ADg-RNA was estimated using the equation $R = 2^{-\Delta Ct}$. These data are summarized graphically using expression values normalized to that detected at 120 h post-transfection.

Supplementary Figure 14

Western blot analysis of hADAR2 expression in tet-ADAR2 cells. hADAR2 and β-actin in lysates from tet-ADAR2 cells cultured in medium containing 0, 0.05, 0.1, 0.5, 1, or 5 μg/ml Dox were analysed by western blotting. Anti-ADARB1 (Sigma) and

anti-actin (Sigma) antibodies were used as primary antibodies for hADAR2 and β-actin, respectively. Detection was achieved using a secondary anti-rabbit IgG antibody conjugated to horseradish peroxidase (Sigma).

Supplementary Figure 15

Change in editing efficiency of FLNA and BLCAP mRNA following Dox-induced ADAR2 expression. Editing efficiencies at the Q/R site in FLNA mRNA and at the Y/C site in BLCAP mRNA were analysed by sequencing after cell culture in the presence or absence of Dox (5 μg/mL). The calculated editing percentages at (**a**) the Q/R and (**b**) Y/C sites are summarized in the bar graph.

Supplementary Figure 16

Analysis of the editing-induction activity of AD-gRNA against GFP mRNA in tet-ADAR2 cells. Editing efficiencies at the A200 site in GFP mRNA extracted from Tet-ADAR2 cells transfected with AD-gRNA expression plasmid. The transfected plasmids included p-5′-AS, p-ADg-GFP_A200, p-ADg-rGFP_A200, p-sADg-GFP_A200, and p-sADg-rGFP_A200. Each editing percentage was calculated

by determining the G/(A + G) peak-height ratio for the A200 site in the sequencing chromatograms.

Supplementary Figure 17

Entire sequencing chromatograms of GFP mRNA extracted from Tet-ADAR2 cells transfected with an AD-gRNA expression plasmid. Entire sequencing chromatograms of GFP cDNA from Tet-ADAR2 cells (**a**) without transfection, or transfected with (**b**) p-5′-AS, (**c**) p-ADg-GFP_A200, (**d**) p-ADg-rGFP_A200, (**e**) p-sADg-GFP_A200, or (**f**) p-sADg-rGFP_A200.

Supplementary Figure 18

Analysis of the effect of AD-gRNA expression on the intracellular editing state in Tet-ADAR2 cells. Editing efficiency at (**a**) the Q/R site in FLNA and (**b**) the Y/C site in BLCAP following transfection with p-5′-AS, p-ADg-GFP_A200, p-ADg-rGFP_A200, p-sADg-GFP_A200, or p-sADg-rGFP_A200

Supplementary Figure 19

Analysis of editing the structured region of GluR2 RNA under general editing reaction conditions. (**a**) GluR2 RNA sequence represented in terms of its predicted secondary structure. (**b**) Sequencing chromatogram of GluR2 RNA after the editing reaction. The structured region corresponding to the ARR site of AD-gRNA is denoted by a bold line.

Supplementary Figure 20

Efficiency of editing exogenously expressed fluorescent reporter RNA and an endogenous mRNA in HEK293 cells transfected with the ADAR2 and AD-gRNA expression plasmids. The editing percentages at (**a**) the A173 site in the GFP-W58X RNA, (**b**) the Q/R site in FLNA mRNA and Y/C site in (**c**) BLCAP mRNA are shown. Editing analysis was performed under the conditions of no AD-gRNA expression plasmid, but with p-5′-AS and p-ADg-rGFP_A173.

Supplementary Figure 21

Entire sequencing chromatograms of fluorescent reporter RNA from HEK293 cells transfected with ADAR2 and the AD-gRNA expression plasmid. Entire

sequencing chromatograms of GFP-W58X RNA are from (**a**) non-transfected HEK293 cells, or p-ADAR2-transfected HEK293 cells (**b**) without co-transfection of an expression plasmid, or (**c**) co-transfected with p-5′-AS or (**d**) p-ADg-rGFP_A173.

Supplementary experimental methods

Gel-mobility shift assay

Annealing was performed as follows. Briefly, 900 nM ADg-GFP_A200 or ADg-rLuc_A311 were annealed with 300 nM sGFP RNA by heating at 80°C for 3 min with subsequent slow cooling to 25°C at 1°C/10 s in annealing buffer (10 mM Tris-HCl [pH 7.6] and 150 mM NaCl). Subsequently, electrophoresis was performed with an 8% polyacrylamide gel, followed by staining with ethidium bromide to detect each band.

RT and qPCR analysis

Subconfluent proliferating Tet-ADAR2 cells were transfected with an AD-gRNA-expression plasmid vector. After culturing for the indicated time, total RNA was extracted and reversed transcribed using random hexamer primers (dN6 primers) with the Transcriptor High Fidelity cDNA Synthesis Kit (Roche). qPCR was performed using the qADg_F and qADg_R primers for specific amplification of ADg-GFP_A200 and the Power SYBR(R) Green PCR Master Mix (Applied Biosystems). Glyceraldehyde 3-phosphate dehydrogenase (GAPDH) was also quantified as an internal standard for quantifying total RNA, using the GAPDH-QF and GAPDH-QR

primers. The qPCR conditions were as follows: denaturation for 10 min at 95°C, and 55 cycles of 15 s at 95°C and 1 min at 60°C, using the LightCycler® Nano system (Roche). Using the LightCycler® Nano analysis software (Roche), Ct values were calculated from the fluorescence-amplification plots by the second-derivative maximum method. The difference in ΔCt was calculated for each sample using the equation ΔCt = Ct (AD-gRNA) − Ct (GAPDH). The relative expression level of ADg-RNA was estimated using the equation R = 2^− Δ Ct. These data are summarized graphically using expression values normalized to that detected at 120 h.

Western blotting analysis for hADAR2 expression in Tet-ADAR2 cells

Tet-ADAR2 cells were cultured in medium containing 0, 0.05, 0.1, 0.5, 1, or 5 μg/mL Dox, and then sub-confluent cells were harvested. After counting with a haemocytometer, 6.4×10^4 cells were lysed in 20 μl SDS-PAGE sample buffer (50 mM Tris-HCl [pH 7.6], 2% SDS, 6% β-mercaptoethanol, 10% glycerol). Proteins in 5 μl samples of each cell lysate were separated by SDS-PAGE using an 8% polyacrylamide gel and electrophoretically transferred to polyvinylidene fluoride (PVDF) membranes (Millipore). To normalize for the amount of lysate loaded, the PVDF membranes were

cut to enable simultaneous analysis of both hADAR2 and β-actin on the same membrane. Each cut membrane was incubated with primary rabbit antibodies against hADAR2 or β-actin (Sigma), and then with a horseradish peroxidase-conjugated secondary antibody against rabbit IgG (Sigma). Following the chemiluminescence reaction with EzWestLumi Plus (ATTO), protein bands were detected by LuminoGraph I (ATTO).

Analysis of the change in editing efficiency in FLNA and BLCAP mRNA following Dox-induced ADAR2 expression

Tet-ADAR2 cells were cultured in 35-mm dishes for 24 h with or without 5 μg/mL Dox. Then, total RNA was extracted and reverse transcribed using the adapter-linked oligo(dT) 17 primer to generate total cDNA, as described in the main text. Using the obtained total cDNA as a template, FLNA and BLCAP cDNA was amplified by PCR with specific primers: (FLNA_F and FLNA_R for FLNA, and BLCAP_F and BLCAP_R for BLCAP). The editing percentages at the Q/R and Y/C sites were analysed by direct sequencing, followed by quantification of the relative heights of the A and G peaks.

Analysis of the effect of AD-gRNA expression on the intracellular editing state in tet-ADAR2 cells

Tet-ADAR2 cells were cultured for 24 h with 5 μg/mL Dox in 35-mm dishes and transfected with 2 μg of each AD-gRNA expression plasmid (p-ADg-GFP_A200, p-ADg-rGFP_A200, p-sADg-GFP_A200, p-sADg-rGFP_A200, or p-5′-AS [as a control]). At 72 h post-transfection, the editing percentages at the Q/R and Y/C sites were analysed as described above. The cells were then transfected with 2 μg of the AD-gRNA expression plasmid, using the X-tremeGENE HP DNA Transfection Reagent (Roche). At 72 h post-transfection, the editing efficiency at A200 in AcGFP RNA, which was simultaneously expressed with hADAR2 in Tet-ADAR2 cells, was analysed as follows. Total RNA was extracted from the transfected cells using Sepasol RNA I Super G (Nacalai Tesque), according to the manufacturer's recommended protocol. Then, RNA samples (30 μg) were treated with 10 U DNase I (Takara Bio) for 1 h at 37ºC, followed by phenol/chloroform extraction and ethanol precipitation. Purified RNA (0.5 μg) was reverse transcribed using the adapter-linked oligo(dT) 17 primer and the Transcriptor High Fidelity cDNA Synthesis Kit (Roche), according to

the manufacturer's recommended protocol. Using the obtained total cDNA as a template, AcGFP cDNA was amplified by PCR with AcGFP-specific primers (AcGFP_F and AcGFP_R). The editing percentages at the Q/R and Y/C sites were analysed by direct sequencing, followed by quantification of the relative heights of the A and G peaks.
