# Supplementary material for: Construction of a guide-RNA for site-directed RNA mutagenesis utilising intracellular A-to-I RNA editing
Source: Sci Rep. 2017 Feb 2;7:41478. doi: 10.1038/srep41478 (PMC5288656; doi:10.1038/srep41478)
Supplement: Supplementary Table [file srep41478-s2.pdf]

Supplementary Table1. Nucleotide sequences of DNA oligonucleotides and primers

for *in vitro* synthesis of ADg-RNA

target	name	sequence (5'→3')
ADg-GFP_A200	ADg-GFP_A200_T7F	CTAATACGACTCACTATAGGGTGGAATAGTATAACAATATGC
	ADg-GFP_A200_RV	TGACCACCCTGAGCTGCGG
ADg-rGFP_A200	ADg-rGFP_A200_T7F	CTAATACGACTCACTATAGGGAAGCACTGCACGCCGCAGCGGGTGGAATAG
	ADg-rGFP_A200_RV01	AGGTGGGATACTATAACAACATTTAGCATATTGTTATACTATTCCACCC
	ADg-rGFP_A200_RV02	AGGTGGGATACTATAACAACATTTAGC
ADg-rRluc_A311	ADg-rRluc_A311_T7F	CTAATACGACTCACTATAGGGTTCAGCAGCTCGAACCAAGGGGTGGAATAGTATAC
	ADg-rRluc_A311_RV	AGGTGGGATACTATACCACGAATGGTATACTATTCCACCC
sADg-GFP_A200	sADg-GFP_A200_T7F	CTAATACGACTCACTATAGGGTGGAATAGTATACCATTCGTGGTATAG
	sADg-GFP_A200_RV	TGACCACCCTGAGCTGCGGAGGTGGGATACTATACCACGAATGG
sADg-rGFP_A200	sADg-rGFP_A200_T7F	CTAATACGACTCACTATAGGGAAGCACTGCACGCCGCAGCGGGTG
	sADg-rGFP_A200_RV	AGGTGGGATACTATACCACGAATGGTATACTATTCCACCCGCTGCGGCG
ADg (L1)	sADg-rGFP_A200_01_T7F	CTAATACGACTCACTATAGGGAAGCACTGCACGCCGCAGTGGGTG
	sADg-rGFP_A200_01_RV	GTAGGTGGGATACTATACCACGAATGGTATACTATTCCACCCACTGCGGCG
ADg (L2)	sADg-rGFP_A200_02_T7F	CTAATACGACTCACTATAGGGAAGCACTGCACGCCGCAGTGGGTG
	sADg-rGFP_A200_02_RV	TAGGTGGGATACTATACCACGAATGGTATACTATTCCACCCACTGCGGCG
ADg (L4)	sADg-rGFP_A200_04_T7F	CTAATACGACTCACTATAGGGAAGCACTGCACGCCGCAGCTGGTG
	sADg-rGFP_A200_04_RV	GGTGGGATACTATACCACGAATGGTATACTATTCCACCAGCTGCGGCG
ADg (L5)	sADg-rGFP_A200_05_T7F	CTAATACGACTCACTATAGGGAAGCACTGCACGCCGCAGCTCGTG
	sADg-rGFP_A200_05_RV	GTGGGATACTATACCACGAATGGTATACTATTCCACGAGCTGCGGCG
sGFP RNA	GFPs RNA_T7F	CTAATACGACTCACTATAGGGTGAATGGCCACAAGTTCAG
	GFPs RNA_RV	TAGCGTGAGAAGCACTGCAC
Rluc W104X RNA	Rluc W104X_Eco_FW	GCTAGGAATTCACCATGGCTTCCAAGGTGTAC
	Rluc W104X_Bam_RV	GAAGGATCCTTACTGCTCGTTCTTC
	Rluc W104X_FW	CTCACCGCTTAGTTCGAGCTG
	Rluc W104X_R01	CAGCTCGAACTAAGCGGTGAG
	Rluc W104X_Koz_T7F	CTAATACGACTCACTATAGGGACCATGGCTTCCAAGGTGTAC
	Rluc W104X_R02	TTACTGCTCGTTCTTCAGCACG

for construction of expression plasmid

target	name	sequence (5'→3')
hADAR2 R1_del	ADAR2_R1del_F	AGTGGGAAGAATCCCGTGATG
hADAR2 R12_del	ADAR2D_ScEx_F	TTGCACTTGGATCAGACGCCATC
	ADAR2_ScEx_XbaR	CGTCTAGATCAGGGCGTGAGTGAGAAC
hADAR2 R2_mut	ADAR2_forR2mut_F	GGCTCGGGGAGAAACGAGGCGCTTGCCGCGGCCCGGGCTGCGC
	ADAR2_fotR2mut_R	GCGCAGCCCGGGCCGCGGCAAGCGCCTCGTTTCTCCCCGAGCC
p-ADg-GFP_A200	ADg-GFP_A200 F1	GGGTGGAATAGTATAACAATATGCTAAATGTTGTTATAGTATCC
	ADg-GFP_A200 R1	TGACCACCCTGAGCTGCGGAGGTGGGATACTATAACAAC
	ADg-GFP_A200 F2	CTAAGATCTGGGTGGAATAGTATAACAATATG
	ADg-GFP_A200 R2	CTAAAGCTTAAAAATGACCACCCTGAGCTGCG
p-ADg-rGFP_A200	ADg-rGFP_A200 F1	GGGAAGCACTGCACGCCGCAGCGGGTGGAATAGTATAACAATATG
	ADg-rGFP_A200 R1	AGGTGGGATACTATAACAACATTTAGCATATTGTTATACTATTC
	ADg-rGFP_A200 F2	CTAAGATCTGGGAAGCACUGCACGCCG
	ADg-rGFP_A200 R2	CTAAAGCTTAAAAAAGGTGGGATACTATAAC
p-sADg-GFP_A200	sADgGFP F	GCTAGAGATCTGGGTGGAATAGTATACCATTCGTG
	sADgGFP R	GCTAGAAGCTTAAAAATGACCACCCTGAGCTG
p-sADg-rGFP_A200	sADgrGFP F	GATAAAGATCTGGGAAGCACTGCACG
	sADgrGFP R	GCTAGAAGCTTAAAAAAGGTGGGATACTATACCACG
p-ADg-rGFP_A173	ADg-rGFP_A173 F	GCTATAGATCTGTCACCAGGGTGGGCCAGGGGGTGGAATAGTATAAC
	ADg-rGFP_A173 R	CCGATAAGCTTAAAAAGGTGGGATACTATAACAACATTTAGCATATTGTTATACTATTCCACCC
p-GFP-W58X	5'-GFP F	GCATGCTCGAGGGGCCGATGGTGAGC
	5'-GFPW58X R	CAGGGTGGGCTAGGGCACAGG
	3'-GFPW58X F	CCTGTGCCCTAGCCCACCCTG

	3'-GFP R	GGTACAAGCTTTCACTTGTACAGCTCATCCA

for RT-PCR and direct sequencing

	name	sequence (5'→3')
RT	oligodT	GGCCACGCGTCGACTAGTACTTTTTTTTTTTTTTTTT
PCR for AcGFP	AcGFP_F	CTAATACGACTCACTATAGGGATGGTGAGCAAGGGCGCC
	AcGFP_R	TCACTTGTACAGCTCATCCA
PCR for FLNA	FLNA_F	
	FLNA_R	
PCR for BLCAP	BLCAP_F	
	BLCAP_R	

for qPCR

target	name	sequence (5'→3')
ADg-GFP_A200	sADgrGFP F for qPCR	GAAGCACTGCACGCCG
	sADgrGFP R for qPCR	GGTGGGATACTATACCACG
GAPDH	GapDH F for qPCR	CCTGCACCACCAACTGCTTAGC
	GapDH R for qPCR	GATGGCATGGACTGTGGTCATGAC

Supplementary Table2. Nucleotide sequences of RNAs used in this study

NAME	Sequence (5'→3')	length (nt)
ADg-GFP_A200 RNA	GGGUGGAAUAGUAUAACAAUAUGCUAAAUGUUGUUAUAGUAUCCCACCUCCGCAGCUCAGGGUGGUCA	68
3'-AS RNA	CCGCAGCUCAGGGUGGUCA	19
ADg-rGFP_A200 RNA	GGGAAGCACUGCACGCCGCAGCGGGUGGAAUAGUAUAACAAUAUGCUAAAUGUUGUUAUAGUAUCCCACCU	71
5'-AS RNA	GGGAAGCACUGCACGCCGCAGC	22
ADg-rRluc_A311 RNA	GGGUUCAGCAGCUCGAACCAAGGGGUGGAAUAGUAUACCAUUCGUGGUAUAGUAUCCCACCU	62
sADg-GFP_A200 RNA	GGGUGGAAUAGUAUACCAUUCGUGGUAUAGUAUCCCACCUCCGCAGCUCAGGGUGGUCA	59
sADg-rGFP_A200 RNA	GGGAAGCACUGCACGCCGCAGCGGGUGGAAUAGUAUACCAUUCGUGGUAUAGUAUCCCACCU	62
sADg-rGFP_A200_0	GGGAAGCACUGCACGCCGCGGUGGGUGGAAUAGUAUACCAUUCGUGGUAUAGUAUCCCACCUACC	65
ADg (L1)	GGGAAGCACUGCACGCCGCAGUGGGUGGAAUAGUAUACCAUUCGUGGUAUAGUAUCCCACCUAC	64
ADg (L2)	GGGAAGCACUGCACGCCGCAGUGGGUGGAAUAGUAUACCAUUCGUGGUAUAGUAUCCCACCUA	63
ADg (L4)	GGGAAGCACUGCACGCCGCAGCUGGUGGAAUAGUAUACCAUUCGUGGUAUAGUAUCCCACC	61
ADg (L5)	GGGAAGCACUGCACGCCGCAGCUCGUGGAAUAGUAUACCAUUCGUGGUAUAGUAUCCCAC	60
ADg-rGFP_A173	GUCACCAGGGUGGGCCAGGGGGUGGAAUAGUAUAACAAUAUGCUAAAUGUUGUUAUAGUAUCCCACCUU	69
AcGFP RNA	AUGGUGAGCAAGGGCGCCGAGCUGUUCACCGGCAUCGUGCCCAUCCUGAUCGAGCUGAAUGGCGAUGUGAAUGGC<br>CACAAGUUCAGCGUGAGCGGCGAGGGCGAGGGCGAUGCCACCUACGGCAAGCUGACCCUGAAGUUCAUCUGCACC<br>ACCGGCAAGCUGCCUGUGCCCUGGCCCACCCUGGUGACCACCCUGAGCUACGGCGUGCAGUGCUUCUCACGCUAC<br>CCCGAUCACAUGAAGCAGCACGACUUCUUCAAGAGCGCCAUGCCUGAGGGCUACAUCCAGGAGCGCACCAUCUUC<br>UUCGAGGAUGACGGCAACUACAAGUCGCGCGCCGAGGUGAAGUUCGAGGGCGAUACCCUGGUGAAUCGCAUCGAG<br>CUGACCGGCACCGAUUUCAAGGAGGAUGGCAACAUCCUGGGCAAUAAGAUGGAGUACAACUACAACGCCCACAAUG<br>UGUACAUCAUGACCGACAAGGCCAAGAAUGGCAUCAAGGUGAACUUCAAGAUCCGCCACAACAUCGAGGAUGGCAG<br>CGUGCAGCUGGCCGACCACUACCAGCAGAAUACCCCCAUCGGCGAUGGCCCUGUGCUGCUGCCCGAUAACCACUA<br>CCUGUCCACCCAGAGCGCCCUGUCCAAGGACCCCAACGAGAAGCGCGAUCACAUGAUCUACUUCGGCUUCGUGAC<br>CGCCGCCGCCAUCACCCACGGCAUGGAUGAGCUGUACAAGUGA	720
GFPs RNA	GGGUGAAUGGCCACAAGUUCAGCGUGAGCGGCGAGGGCGAGGGCGAUGCCACCUACGGCAAGCUGACCCUGAAGU<br>UCAUCUGCACCACCGGCAAGCUGCCUGUGCCCUGGCCCACCCUGGUGACCACCCUGAGCUACGGCGUGCAGUGCU<br>UCUCACGCUA	160
Rluc W104X RNA	GGGACCAUGGCUUCCAAGGUGUACGACCCCGAGCAACGCAAACGCAUGAUCACUGGGCCUCAGUGGUGGGCUCGC<br>UGCAAGCAAAUGAACGUGCUGGACUCCUUCAUCAACUACUAUGAUUCCGAGAAGCACGCCGAGAACGCCGUGAUUU<br>UUCUGCAUGGUAACGCUGCCUCCAGCUACCUGUGGAGGCACGUCGUGCCUCACAUCGAGCCCGUGGCUAGAUGCA<br>UCAUCCCUGAUCUGAUCGGAAUGGGUAAGUCCGGCAAGAGCGGGAAUGGCUCAUAUCGCCUCCUGGAUCACUACA<br>AGUACCUCACCGCUUAGUUCGAGCUGCUGAACCUUCCAAAGAAAAUCAUCUUUGUGGGCCACGACUGGGGGGCUU<br>GUCUGGCCUUUCACUACUCCUACGAGCACCAAGACAAGAUCAAGGCCAUCGUCCAUGCUGAGAGUGUCGUGGACG<br>UGAUCGAGUCCUGGGACGAGUGGCCUGACAUCGAGGAGGAUAUCGCCCUGAUCAAGAGCGAAGAGGGCGAGAAAA<br>UGGUGCUUGAGAAUAACUUCUUCGUCGAGACCAUGCUCCCAAGCAAGAUCAUGCGGAAACUGGAGCCUGAGGAGUU<br>CGCUGCCUACCUGGAGCCAUUCAAGGAGAAGGGCGAGGUUAGACGGCCUACCCUCUCCUGGCCUCGCGAGAUCCC<br>UCUCGUUAAGGGAGGCAAGCCCGACGUCGUCCAGAUUGUCCGCAACUACAACGCCUACCUUCGGGCCAGCGACGA<br>UCUGCCUAAGAUGUUCAUCGAGUCCGACCCUGGGUUCUUUUCCAACGCUAUUGUCGAGGGAGCUAAGAAGUUCCCU<br>AACACCGAGUUCGUGAAGGUGAAGGGCCUCCACUUCAGCCAGGAGGACGCUCCAGAUGAAAUGGGUAAGUACAUC<br>AAGAGCUUCGUGGAGCGCGUGCUGAAGAACGAGCAGUAA	942
AcGFP W58X RNA	AUGGUGAGCAAGGGCGCCGAGCUGUUCACCGGCAUCGUGCCCAUCCUGAUCGAGCUGAAUGGCGAUGUGAAUGGC<br>CACAAGUUCAGCGUGAGCGGCGAGGGCGAGGGCGAUGCCACCUACGGCAAGCUGACCCUGAAGUUCAUCUGCACC<br>ACCGGCAAGCUGCCUGUGCCCUAGCCCACCCUGGUGACCACCCUGAGCUACGGCGUGCAGUGCUUCUCACGCUAC<br>CCCGAUCACAUGAAGCAGCACGACUUCUUCAAGAGCGCCAUGCCUGAGGGCUACAUCCAGGAGCGCACCAUCUUC<br>UUCGAGGAUGACGGCAACUACAAGUCGCGCGCCGAGGUGAAGUUCGAGGGCGAUACCCUGGUGAAUCGCAUCGAG<br>CUGACCGGCACCGAUUUCAAGGAGGAUGGCAACAUCCUGGGCAAUAAGAUGGAGUACAACUACAACGCCCACAAUG<br>UGUACAUCAUGACCGACAAGGCCAAGAAUGGCAUCAAGGUGAACUUCAAGAUCCGCCACAACAUCGAGGAUGGCAG<br>CGUGCAGCUGGCCGACCACUACCAGCAGAAUACCCCCAUCGGCGAUGGCCCUGUGCUGCUGCCCGAUAACCACUA<br>CCUGUCCACCCAGAGCGCCCUGUCCAAGGACCCCAACGAGAAGCGCGAUCACAUGAUCUACUUCGGCUUCGUGAC<br>CGCCGCCGCCAUCACCCACGGCAUGGAUGAGCUGUACAAGUGA	720
